# Application of anti-inflammatory treatment in two different ovine Acute Respiratory Distress Syndrome injury models: a preclinical randomized intervention study

**DOI:** 10.1038/s41598-023-45081-8

**Published:** 2023-10-20

**Authors:** Karin Wildi, Samantha Livingstone, Carmen Ainola, Sebastiano Maria Colombo, Silver Heinsar, Noriko Sato, Kei Sato, Mahé Bouquet, Emily Wilson, Gabriella Abbate, Margaret Passmore, Kieran Hyslop, Keibun Liu, Xiaomeng Wang, Chiara Palmieri, Louise E. See Hoe, Jae-Seung Jung, Katrina Ki, Christian Mueller, John Laffey, Paolo Pelosi, Gianluigi Li Bassi, Jacky Suen, John Fraser

**Affiliations:** 1 Critical Care Research Group, The Prince Charles Hospital, Brisbane, Australia; 2 The University of Queensland, Brisbane, Australia; 3 Cardiovascular Research Institute Basel, University Hospital Basel, University of Basel, Basel, Switzerland; 4 Department of Anaesthesia and Intensive Care Medicine, Fondazione IRCCS Ca’ Granda Ospedale Maggiore Policlinico, Milan, Italy; 5 Center for Cardiac Intensive Care, Beijing Anzhen Hospital, Capital Medical University, Beijing, People’s Republic of China; 6 The University of Queensland, School of Veterinary Science, Gatton, Australia; 7 Department of Thoracic and Cardiovascular Surgery, College of Medicine, Korea University, Seoul, Republic of Korea; 8 Galway University Hospitals, University of Galway, Galway, Ireland; 9 Anesthesiology and Critical Care, San Martino Policlinico Hospital, IRCCS for Oncology and Neurosciences, Genoa, Italy; 10 Department of Surgical Sciences and Integrated Diagnostics, University of Genoa, Genoa, Italy; 11 Queensland University of Technology, Brisbane, Australia; 12 Uniting Care Hospitals, St Andrews War Memorial and The Wesley Intensive Care Units, Brisbane, Australia

**Keywords:** Respiratory tract diseases, Translational research, Diseases

## Abstract

Whilst the presence of 2 subphenotypes among the heterogenous Acute Respiratory Distress Syndrome (ARDS) population is becoming clinically accepted, subphenotype-specific treatment efficacy has yet to be prospectively tested. We investigated anti-inflammatory treatment in different ARDS models in sheep, previously shown similarities to human ARDS subphenotypes, in a preclinical, randomized, blinded study. Thirty anesthetized sheep were studied up to 48 h and randomized into: (a) OA: oleic acid (n = 15) and (b) OA-LPS: oleic acid and subsequent lipopolysaccharide (n = 15) to achieve a PaO_2_/FiO_2_ ratio of < 150 mmHg. Then, animals were randomly allocated to receive treatment with methylprednisolone or erythromycin or none. Assessed outcomes were oxygenation, pulmonary mechanics, hemodynamics and survival. All animals reached ARDS. Treatment with methylprednisolone, but not erythromycin, provided the highest therapeutic benefit in Ph2 animals, leading to a significant increase in PaO_2_/FiO_2_ ratio by reducing pulmonary edema, dead space ventilation and shunt fraction. Animals treated with methylprednisolone displayed a higher survival up to 48 h than all others. In animals treated with erythromycin, there was no treatment benefit regarding assessed physiological parameters and survival in both phenotypes. Treatment with methylprednisolone improves oxygenation and survival, more so in ovine phenotype 2 which resembles the human hyperinflammatory subphenotype.

## Introduction

Acute Respiratory Distress Syndrome (ARDS) is a life-threatening syndrome caused by a large variety of etiologies^1^. Although a large body of data has characterized critical features of such syndrome^2,3^, its broad etiological heterogeneity and variable host-responses have impeded large improvements in patient mortality^4^ and morbidity rates^5^.

Based on the presumed predominant inflammatory pathogenesis in ARDS, treatment with corticosteroids in ARDS has been studied extensively in the past decades^6^, but corticosteroids are currently not recommended due to the conflicting results^7,8^. It has been suggested that potential beneficial effects of corticosteroids may have been offset due to its application in highly heterogenous populations. Indeed, post-hoc analyses of clinical studies revealed evidence for distinct ARDS subpenotypes: a hypoinflammatory and a hyperinflammatory subphenotype^9–13^. Briefly, the latter is characterized by a more severe inflammatory state, causing hemodynamic derangement and non-pulmonary organ failure, as well as higher mortality^9–13^. Importantly, retrospective post-hoc analyses of randomized clinical trials provided insightful suggestions on the risk of negative results when potentially beneficial treatments are applied to populations with mixed ARDS subphenotypes^9,11,12,14^. These retrospective findings have not been corroborated yet in prospective subphenotype-specific populations, due to the difficulties in accurately define these populations.

In this context, large animal models of ARDS subphenotypes could further elucidate benefits and harms of proposed interventions^15^ and inform the design of future innovative clinical studies. We previously assessed models of lung injury in sheep, and demonstrated that the double-hit ARDS model (oleic acid and lipopolysaccharides (OA-LPS)) mimics key features as observed in the human hyperinflammatory subphenotype^16^. Additionally, a recent report^17^ demonstrated comparable gene expression in tracheal aspirate among LPS animal models and human hyperinflammatory subphenotype. OA alone was chosen as the opposite ARDS lung injury model with the least systemic inflammation activation as compared to OA-LPS^16^.

We hypothesized that a correct selection of injury model may result in early clinical improvement in ovine ARDS. Thus, we investigated the effects of corticosteroids and macrolides on oxygenation (1° outcome) and lung edema, pulmonary mechanics, hemodynamic parameters and survival (2° outcome) in aforementioned sheep models of ARDS phenotypes.

## Methods

Animal studies were conducted at the Queensland University of Technology (QUT) Medical Engineering Facility (MERF) in Brisbane. Animal ethics was approved by QUT Office of Research Ethics and Integrity (No 18-606). All experiments were performed in accordance with the Australian Code of Practice for the Care and Use of Animals for Scientific Purposes and the Animal care and Protection Act 2001 (QLD) and complied with the ARRIVE Guidelines.

### Study design

**Figure 1:**
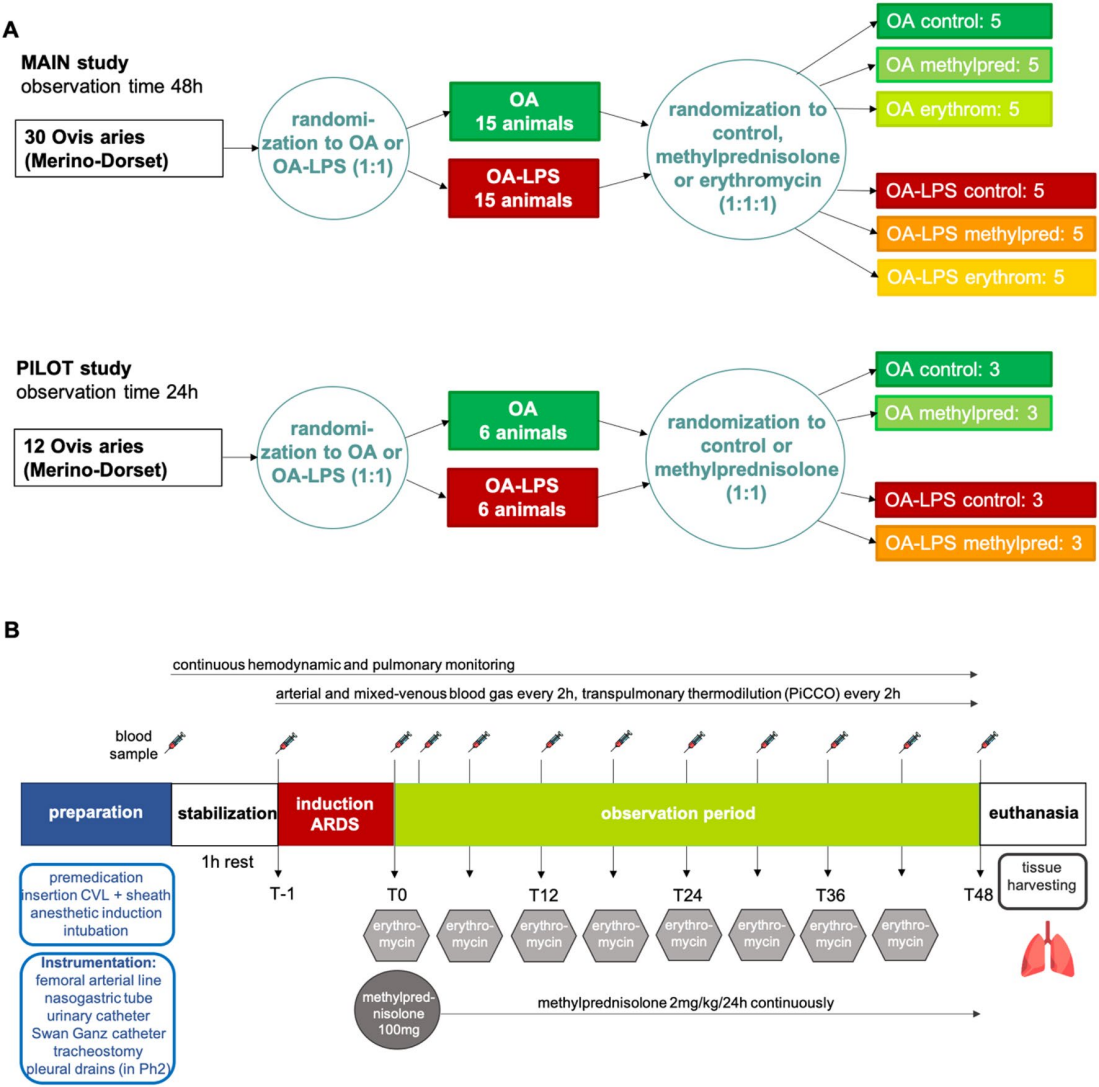
(**A**) study design of STARDUST and (**B**) timeline of the experiment**.** Abbreviations: ARDS: Acute Respiratory Distress Syndrome; CVL: central venous line; PiCCO: pulse-contour cardiac output; OA: Oleic Acid; OA-LPS: Oleic Acid and lipopolysaccharides; PF ratio: PaO_2_/FiO_2_ ratio.

This was a single-blinded, randomized, controlled preclinical trial in an ovine model (Fig. 1A). A total of 30 animals were randomized and allocated to the two injury methods: oleic acid (OA, n = 15) or oleic acid and lipopolysaccharides (OA-LPS, n = 15). For each injury model, subjects were further randomized to receive treatment either with methylprednisolone (pred: OA-LPS-pred, OA-pred; each 5 per group) or erythromycin (ery: OA-LPS-ery, OA-ery; each 5 per group) or they were randomized to the control group (ctr: OA-LPS-ctr and OA-ctr; each 5 per group). A pilot study consisting of 12 animals was conducted to confirm safety, adherence to protocol and efficacy assumptions in relation to treatment (Fig. 1A): animals were randomized to either pred or ctr per each phenotype, resulting in 3 animals per group. Randomization was performed using a random number generator.

### Animal preparation

All sheep were approved for use by the facility veterinarian after a comprehensive health check and full blood count. Prior to experimentation, all animals were fasted overnight with free access to drinking water. A four-lumen central venous catheter (CVL; Arrow Int., Reading, USA) and a venous sheath (Edwards Lifesciences, Irvine, USA) were inserted in the jugular veins via Seldinger technique and sutured in place. Cefazolin 1 g intravenously (IV) was administered for antibiotic prophylaxis. For induction of anesthesia, midazolam (0.5 mg/kg) and propofol (3–4 mg/kg) were administered IV. A cuffed endotracheal tube size 9.0 internal diameter (Mallinckrodt, USA) was inserted into the airway, and mechanical ventilation (Galileo 5, Hamilton Medical, Switzerland) was commenced after placing the animal on the operative table in supine position. Initial ventilation settings consisted of tidal volumes of 8 ml/kg, a PEEP of 5 cmH_2_0, a respiratory rate of 12–16 breaths/min and an FiO_2_ of 30–50%; settings were adapted to maintain an arterial saturation of > 94% and normocapnia, defined by an end-tidal carbon dioxide (EtCO_2_) of 35–45 mmHg. General anesthesia was maintained and titrated to an appropriate level with continuous infusion of midazolam 0.5–0.8 mg/kg/h, fentanyl 5–20 µg/kg/h and ketamine 2.5–7.5 mg/kg/h. Basic monitoring consisted of a pulse oximeter probe attached to the tongue, a 5-lead electrocardiogram and continuous waveform capnography. A PiCCO catheter (PULSION Medical Systems, Getinge, Gothenburg, Sweden) was inserted into the femoral artery for advanced hemodynamic monitoring. A pulmonary artery catheter (Swan-Ganz CCOmbo, Edwards Lifescience, Irvine, USA) was inserted via the internal jugular sheath to monitor pulmonary artery pressure, mixed-venous saturation and core body temperature. In addition, a 14F nasogastric tube was placed and left on free drainage, and a 12F urinary catheter was inserted with closed collection. To facilitate access to the airways for bronchoscopy and decrease dead space ventilation, a surgical tracheotomy with insertion of a cuffed endotracheal tube (Size 9–10, Portex) was performed. During instrumentation, a crystalloid bolus (Hartmanns) of 250 ml was delivered to compensate for overnight fasting, then the fluids infusion rate was maintained at 1 ml/kg/h and modified as according to the hemodynamic condition. After instrumentation, the animal was positioned in prone position and rested for one hour prior to commencement of induction of ARDS (Fig. 1B).

### Induction of ARDS injury models

Using the official ARDS definition for experimental models^18,19^, animals randomized to OA received sequential IV administration of oleic acid (0.89 g/ml; Sigma-Aldrich, Australia) in subsequent 0.03 ml/kg doses to ultimately achieve a PaO_2_/FiO_2_ < 150, as assessed via arterial blood gases test 15 min after OA infusion^20^. OA-LPS group received administration of OA, then after fulfilling ARDS criteria, 0.5 µg/kg of lipopolysaccharide (LPS: *E. coli* O55:B5, Sigma-Aldrich, Australia), dissolved in 50 ml of normal saline, was infused over 1 h.

### Anti-inflammatory treatment and blinding

Animals randomized to treatment with methylprednisolone received a bolus of 100 mg IV at confirmation of ARDS diagnosis (T0) and a continuous infusion of 2 mg/kg/24 h over the course of the study (Fig. 1B). Animals randomized to treatment with erythromycin received a bolus of 100 mg IV every 6 h, starting at T0. Investigators involved in the clinical management of the study subjects were blinded to treatment allocation: all animals received two 10 ml boluses at T0 (one containing methylprednisolone 100 mg or normal saline (NS), and another containing erythromycin 100 mg or NS), followed by a continuous infusion of 2 ml/h (containing methylprednisolone 2 mg/kg/24 h or NS) and a 10 ml bolus every 6 h (containing erythromycin 100 mg or NS).

### Intra-experimental monitoring and management

Arterial blood gas analysis was performed every 2 h and whenever clinically indicated to assess blood gases, glucose and electrolytes. Mixed-venous blood gas analysis and transpulmonary thermodilution using Pulse Contour Cardiac Output (PiCCO) technology was performed every 2 h (Fig. 1B). Intra-experimental monitoring and data recording are described in the Supplemental Methods.

To minimize potential confounders, strict protocols for hemodynamic management (Supplemental Fig. S1), ventilation strategy (Supplemental Fig. S2) and procedures were applied.

Animals were euthanized with 163 mg/kg of pentobarbital and organs were retrieved for tissue harvesting at the end of the study, 48 h after development of ARDS, or when animals reached the following criteria: (a) mean arterial blood pressure (MAP) persistently below 40 mmHg despite optimized support; and (b) arterial pH below 7.0 because of respiratory, metabolic or mixed acidosis and exhausted compensation strategies.

### Primary and secondary outcomes

The primary outcome was oxygenation (PaO_2_/FiO_2_ ratio (PF ratio) and Oxygenation Index (OI)) throughout the 48 h assessment period among injury types and their respective treatment. As secondary outcomes, we investigated the effects of study treatment on lung edema (extravascular lung water index (EVLWI)), hemodynamic parameters, pulmonary mechanics, and survival in the respective lung injury.

### Sample collection and processing

Blood samples for full blood count (Mindray Hematology analyzer BC 5000, China) and biochemistry (IDEXX laboratories Brisbane, Australia) were collected at baseline and every 12 h following T0. Postmortem collected lung tissue samples were prepared for histopathology. Quantification of pulmonary edema was determined by measuring the wet-to-dry-weight of post-mortem left and right upper and lower lung lobe: a tissue sample of 2 cm diameter and 2 cm thickness was excised and lung weight measured immediately. Dry weight was determined by placing the lung tissue in an oven at 65 °C for several days, weighing the tissue every day until the weight stopped decreasing. The wet-to-dry weight ratio was calculated by dividing the wet by the dry weight.

### Inflammatory cytokines and histopathology

Arterial blood samples were collected in EDTA blood tubes, processed to plasma and stored at − 80 °C until analysis. Plasma concentration of inflammatory cytokines (e.g. interleukin (IL) -6, -8, -10, -1β) in serum were quantified by in-house ELISA’s^21^.

At study end, lung tissues were taken for histological assessment (Suppl. methods). Slides were examined by blinded, qualified veterinary pathologist. The lung injury score (LIS) was assessed as recommended by the ATS for experimental ARDS in animal models^18,19^: it scores neutrophils in the alveolar space (A) and in the interstitial space (B), hyaline membranes (C), proteinaceous debris filling the airspace (D) and alveolar septal thickening (E). Every item is given a score between 0 to 2, then the LIS is calculated by: ((20 × A) + (14 × B) + (7 × C) + (7 × D) + (2 × E))/number of fields × 100. This results in a score between 0 (no injury) and 1 (severe lung injury).

Twenty random high-power fields (400 × total magnification) were scored per section and the LIS was calculated per animal (mean ± SD). For the scoring, at least 50% of each field had to be filled by lung alveoli: fields consisting predominantly of the lumen of large airways or vessels were rejected. Septal thickness was not evaluated in alveolar septa directly adjacent to a blood vessel or airway (normally thickened by the collagen present in the peribroncho-vascular bundle). In addition, the following parameters were evaluated: (a) percentage of section effaced by necrosis (score/field: 0 = none or less than 10%; 1 = 10–50%; 2 =  > 50%) and (b) number of thrombi within blood vessels (score/field: 0 = none; 1 = 1 thrombus; 2 =  > 1 thrombus). Bacterial colonies as observed in necrotic areas or admixed with the neutrophilic aggregates and alveolar hemorrhage were marked as present or not within one animal and reported as percentage per injury type and the respective treatment group.

### Statistical analysis

For the power calculation, an F-test for correction to 6 treatment groups was applied (power set at 80%, level of α at 5%, 2-tailed), assuming a treatment effect in terms of an improvement in PaO_2_/FiO_2_ ratio of 80 mmHg and a standard deviation (SD) of 40 mmHg^22^ over the course of the study. Conservatively assuming a Cohens d of 1.0 (medium effect size), this resulted in a total sample size of 28 animals, therefore 5 animals per group.

Categorical variables are reported as numbers and percentages, continuous data as mean ± SD or median and interquartile range (IQR), for normally or not-normally distributed parameters, respectively. Comparisons between groups were made using Kruskal–Wallis or Mann–Whitney U test and x^2^ test as appropriate.

Linear-mixed effects models (LMM)^23^ were constructed to assess the impact of parameters of oxygenation, hemodynamics, metabolic situation and respiratory mechanics over time among injury types and their treatment. Distribution of data was assessed with QQ and residual plots. Control animals were defined as the reference level, time was specified as a continuous fixed effect and the interaction between treatment and time was included. A random effect was defined for each individual to account for within-subject correlation from repeated measurements over time. Fixed effects were reported as estimates with 95% confidence intervals (CI) and p-values were estimated using Sattherwaite’s method.

The pairwise comparison of injury types (2 groups) over time was performed using averaging over time and injury type, the Kenward-Roger method was applied for the degrees of freedom.

A shared parameter joint model^24^ for assessment of longitudinal and time-to-event data (death) and the associated treatment effect was estimated under a Bayesian framework. The model uses observed longitudinal measurement (trajectories) to determine the posterior probability for event (death) and treatment effect for each of the assessed parameter. Data were reported as hazard ratio (HR) and 95% credible intervals.

All statistical analyses were performed with R Version 4.0.5 (R Foundation for Statistical Computing, Vienna, Austria). The minimal reproducible code for the LMM and Bayesian model is reported in the Supplemental Methods.

## Results

### Studied population

A total of 30 female non-pregnant Merino-Dorset crossbreed ewes, aged 1–3 years, mean weight 51 ± 5 kg, were included in the final analysis (Fig. 1A). Baseline characteristics of animals are shown in Supplemental Table S1.

All animals reached ARDS criteria within 2–3 h, median PF ratio at T0 was 138 (IQR 120–149). ARDS was achieved through a median dose of OA of 0.14 ml/kg (IQR 0.012–0.18 ml/kg) with no significant difference among the six groups.

**Figure 2:**
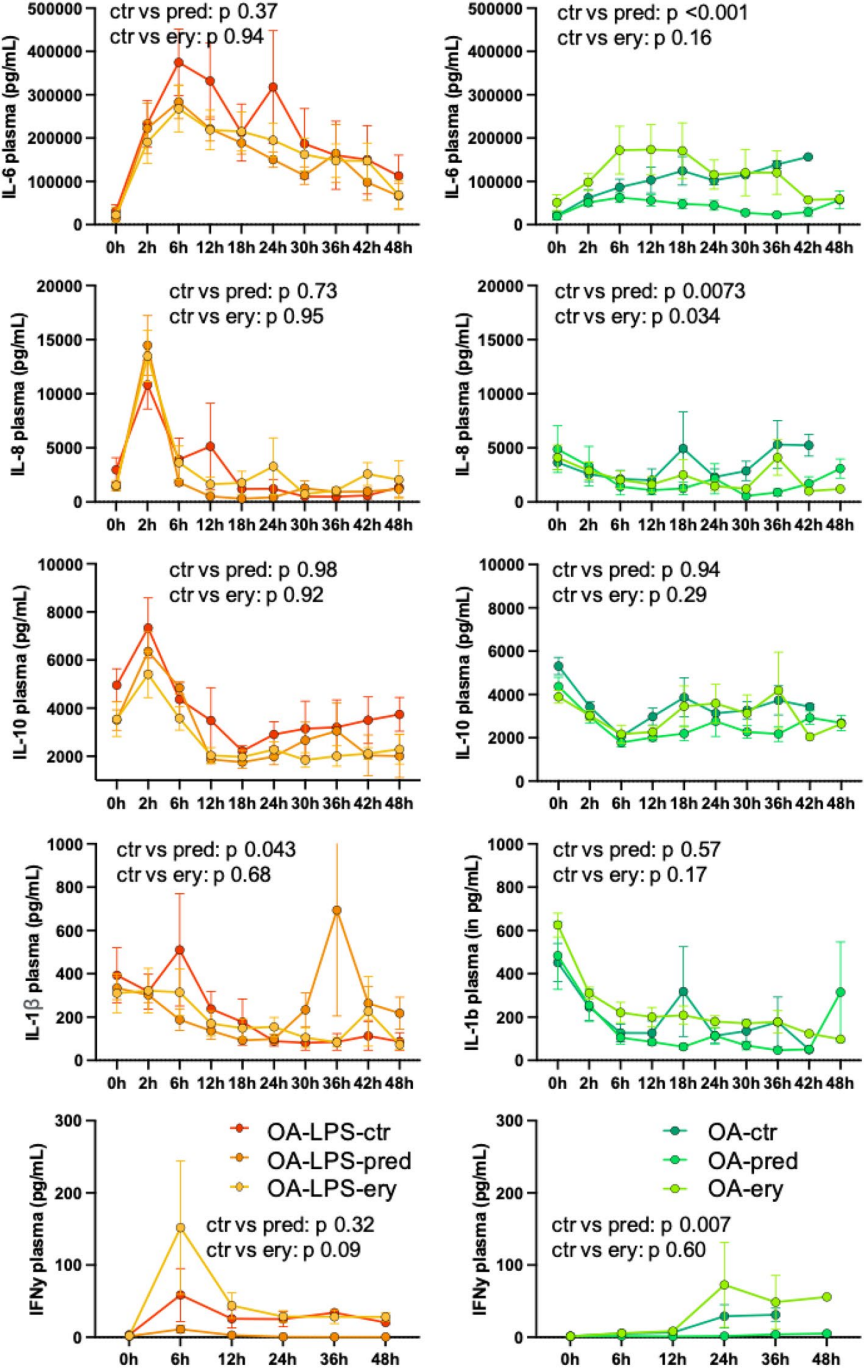
Cytokine levels among all OA-LPS and OA animals in plasma. P values denotes interaction of treatment group over time from linear-mixed effect model. Abbreviations: ctr: control; ery: erythromycin; IFNy: interferon gamma; IL: interleukin; OA: Oleic Acid; OA-LPS: Oleic Acid and lipopolysaccharides; pred: methylprednisolone.

In terms of inflammatory reaction, OA-LPS groups showed higher levels of IL-6, IL-8, IL-10 and IFNy as compared to OA groups early after ARDS induction with a peak at 2 to 6 h (Fig. 2). However, there was no systematic difference over time among the treatment groups in both injury types.

### Primary outcome

**Figure 3:**
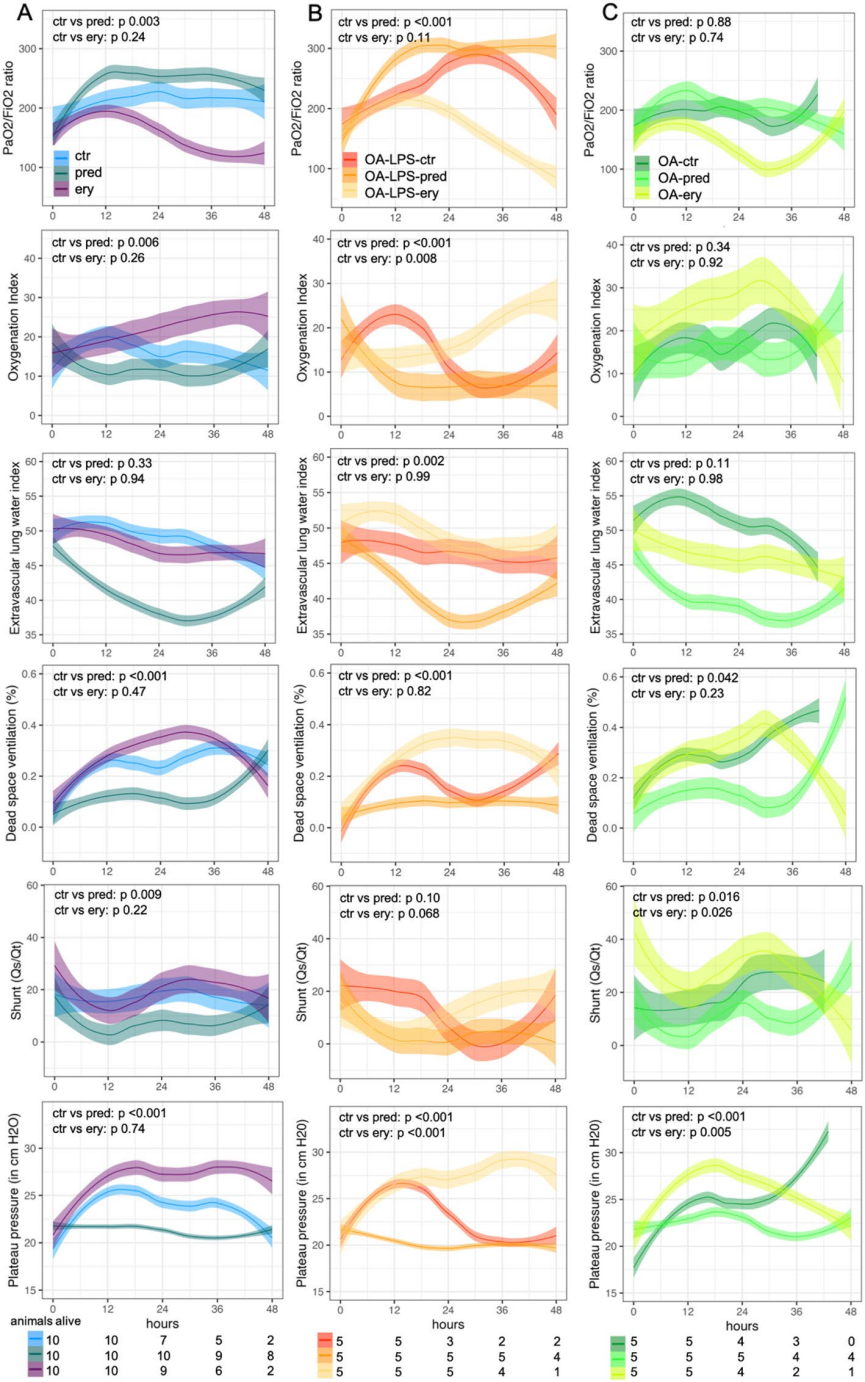
Oxygenation and pulmonary derangement among (**A**) treatment groups (injury types combined) (**B**) treatment among OA-LPS and (**C**) treatment among OA; displayed in mean over time of observation. Curve smoothened by using the LOESS method (locally estimated scatterplot smoothing), p-values for interaction of treatment group over time. Abbreviations: ctr: control; ery: erythromycin; OA: Oleic Acid; OA-LPS: Oleic Acid and lipopolysaccharides; pred: methylprednisolone; Qs/Qt: shunt.

Treatment with methylprednisolone resulted in a higher PF ratio and a significantly lower OI, while animals treated with erythromycin displayed worse oxygenation than control animals (p for interaction (IA) treatment:time < 0.05) (Fig. 3A; Table 1). Clustering between injury type and treatment confirmed a significantly improved PF ratio and OI among OA-LPS in animals treated with methylprednisolone (p for IA treatment:time < 0.001) and a worse oxygenation in animals treated with erythromycin as compared to controls (Fig. 3B; Table 1). Among OA, no significant difference between treatment groups over time were seen (p for IA ns) (Fig. 3C, Table 1). Analyzing OA and OA-LPS independently of treatment (ctr, pred and ery combined), PF ratio and OI did not differ between injury models (Supplemental Fig. S3 and Supplemental Table S2).

**Table 1:** Linear mixed-effect model for control, treatment with methylprednisolone or erythromycin among injury models.

		OA and OA-LPS combined	OA-LPS	OA
		Estimates (95% CI), p value	Estimates (95% CI), p value	Estimates (95% CI), p value
PaO_2_/FiO_2_ ratio (PF ratio)				
Treatment group	Control	Reference	Reference	Reference
	Methylprednisolone	18.7 (− 44.1 to 81.5), 0.57	13.5 (− 72.2 to 99.1), 0.77	22.4 (− 59.6 to 106.3), 0.61
	Erythromycin	− 12.8 (− 75.8 to 50.2), 0.70	1.4 (− 84.3 to 87.1), 0.98	− 27.4 (− 110.8 to 56.0), 0.55
Time, hours		− 1.0 (− 1.8 to − 0.2), 0.01	− 0.6 (− 1.5 to 0.4), 0.24	− 1.5 (− 2.6 to 0− .3), 0.01
Interaction between treatment group and time	Control	Reference	Reference	Reference
	Methylprednisolone	1.4 (0.5 to 2.3), 0.003	2.8 (1.6 to 3.9), < 0.001	− 0.1 (− 1.5 to 1.3), 0.88
	Erythromycin	− 0.6 (− 1.6 to 0.4), 0.24	− 1.0 (− 2.2 to 0.2), 0.11	− 0.3 (− 1.9 to 1.3), 0.74
Oxygenation Index (OI)				
Treatment group	Control	Reference	Reference	Reference
	Methylprednisolone	− 3.8 (− 16.1 to 8.4), 0.55	− 5.4 (− 19.6 to 8.6), 0.48	− 1.8 (− 21.5 to 18.0), 0.89
	Erythromycin	− 1.0 (− 13.3 to 11.3), 0.88	− 9.4 (− 23.5 to 4.7), 0.24	7.5 (− 12.4 to 27.3), 0.49
Time, hours		0.3 (0.2 to 0.4), < 0.001	0.1 (− 0.01 to 0.26), 0.06	0.5 (0.2 to 0.7), < 0.001
Interaction between treatment group and time	Control	Reference	Reference	Reference
	Methylprednisolone	− 0.2 (− 0.4 to − 0.06), 0.006	− 0.3 (− 0.5 to − 0.1), < 0.001	− 0.1 (− 0.4 to 0.1), 0.34
	Erythromycin	0.1 (− 0.007 to 0.3), 0.26	0.2 (0.1 to 0.4), 0.008	− 0.02 (− 0.3 to 0.3), 0.92
Extravascular Lung Water Index (EVLWI)				
Treatment group	Control	Reference	Reference	Reference
	Methylprednisolone	− 7.8 (− 13.1 to − 2.4), 0.009	− 3.0 (− 11.3 to 5.3), 0.51	− 12.6 (− 19.5 to − 5.6), 0.0047
	Erythromycin	− 1.2 (− 6.6 to 4.2), 0.68	63.2 (− 5.1 to 11.4), 0.48	− 5.4 (− 12.4 to 1.5), 0.17
Time, hours		− 0.09 (− 0.1 to − 0.03), 0.002	− 0.08 (− 0.1 to − 0.004), 0.042	− 0.1 (− 0.2 to − 0.02), 0.014
Interaction between treatment group and time	Control	Reference	Reference	Reference
	Methylprednisolone	− 0.03 (− 0.1 to 0.03), 0.33	− 0.1 (− 0.2 to − 0.05), 0.002	0.08 (− 0.02 to 0.2), 0.11
	Erythromycin	0.003 (− 0.07 to 0.07), 0.94	− 0.1 (− 0.2 to 0.09), 0.99	− 0.002 (− 0.1 to 0.1), 0.98
Dead space ventilation (%)				
Treatment group	Control	Reference	Reference	Reference
	Methylprednisolone	− 25.8 (− 81.7 to 30.0), 0.38	− 16.3 (− 92.1 to 59.5), 0.69	− 0.1 (− 0.3 to 0.06), 0.22
	Erythromycin	23.4 (− 32.5 to 79.4), 0.42	10.7 (− 65.2 to 86.6), 0.79	0.04 (− 0.2 to 0.2), 0.73
Time, hours		3.0 (2.5 to 3.6), < 0.001	2.7 (2.0 to 3.3), < 0.001	0.007 (0.006 to 0.01), < 0.001
Interaction between treatment group and time	Control	Reference	Reference	Reference
	Methylprednisolone	− 1.7 (− 2.4 to − 1.0), < 0.001	− 2.2 (− 2.9 to − 1.4), < 0.001	− 0.001 (− 0.004 to 0.002), 0.42
	Erythromycin	− 0.3 (− 1.0 to 0.5), 0.47	0.1 (− 0.7 to 1.0), 0.82	− 0.002 (− 0.005 to 0.001), 0.23
Shunt (Qs/Qt)				
Treatment group	Control	Reference	Reference	Reference
	Methylprednisolone	− 4.6 (− 18.5 to 9.2), 0.53	− 4.6 (− 19.1 to 9.9), 0.56	− 7.5 (− 28.0 to 13.0), 0.51
	Erythromycin	4.0 (− 9.8 to 17.8), 0.58	− 10.8 (− 25.2 to 3.6), 0.18	16.9 (− 3.5 to 37.3), 0.14
Time, hours		0.5 (0.2 to 0.7), < 0.001	0.08 (− 0.2 to 0.3), 0.51	0.7 (0.4 to 1.1), < 0.001
Interaction between treatment group and time	Control	Reference	Reference	Reference
	Methylprednisolone	− 0.4 (− 0.6 to − 0.08), 0.009	− 0.2 (− 0.5 to 0.07), 0.10	− 0.3 (− 0.7 to 0.1), 0.16
	Erythromycin	− 0.2 (− 0.5 to 0.1), 0.22	0.3 (− 0.005 to 0.6), 0.068	− 0.6 (− 1.0 to − 0.07), 0.026
Plateau pressure (cm H_2_0)				
Treatment group	Control	Reference	Reference	Reference
	Methylprednisolone	− 0.9 (− 4.9 to 3.1), 0.67	− 3.6 (− 8.5 to 1.3), 0.19	2.0 (− 4.2 to 8.3), 0.56
	Erythromycin	1.6 (− 2.4 to 5.5), 0.46	− 0.8 (− 5.7 to 4.1), 0.77	4.0 (− 2.3 to 10.2), 0.26
Time, hours		0.2 (0.1 to 0.2), < 0.001	0.06 (0.02 to 0.09), < 0.001	0.3 (0.2 to 0.3), < 0.001
Interaction between treatment group and time	Control	Reference	Reference	Reference
	Methylprednisolone	− 0.2 (− 0.2 to − 0.1), < 0.001	− 0.09 (− 0.1 to − 0.04), < 0.001	− 0.2 (− 0.3 to − 0.2), < 0.001
	Erythromycin	− 0.006 (− 0.04 to 0.02), 0.74	0.08 (0.03 to 0.1), < 0.001	− 0.08 (− 0.1 to − 0.02), 0.005
Mean arterial pressure (mmHg)				
Treatment group	Control	Reference	Reference	Reference
	Methylprednisolone	6.1 (1.1 to11.1), 0.026	3.9 (− 2.3 to 10.1), 0.26	7.8 (0.3 to 15.4), 0.07
	Erythromycin	− 4.8 (− 9.8 to 0.3), 0.078	− 7.5 (− 13.7 to − 1.3), 0.038	− 2.0 (− 9.6 to 5.6), 0.63
Time, hours		− 0.3 (− 0.3 to − 0.2), < 0.001	− 0.06 (− 0.2 to 0.07), 0.36	− 0.5 (− 0.6 to − 0.4), < 0.001
Interaction between treatment group and time	Control	Reference	Reference	Reference
	Methylprednisolone	0.1 (0.02 to 0.2), 0.014	0.07 (− 0.08 to 0.2), 0.33	0.2 (0.04 to 0.3), 0.07
	Erythromycin	0.2 (0.09 to 0.3), < 0.001	0.07 (− 0.09 to 0.2), 0.077	0.3 (0.1 to 0.5), 0.08
Heart rate (bpm)				
Treatment group	Control	Reference	Reference	Reference
	Methylprednisolone	− 22.4 (− 33.9 to − 11.0), < 0.001	− 20.9 (− 35.8 to − 6.0), 0.02	− 23.2 (− 40.4 to − 6.1), 0.02
	Erythromycin	− 4.2 (− 15.7 to 7.3), 0.49	− 9.0 (− 23.9 to 5.9), 0.28	2.0 (− 15.2 to 19.2), 0.83
Time, hours		− 0.4 (− 0.6 to − 0.2), < 0.001	− 0.7 (− 0.9 to − 0.5), < 0.001	− 0.05 (− 0.3 to 0.2), 0.68
Interaction between treatment group and time	Control	Reference	Reference	Reference
	Methylprednisolone	− 0.4 (− 0.6 to − 0.2), < 0.001	− 0.2 (− 0.4 to 0.08), 0.18	− 0.6 (− 0.8 to − 0.3), < 0.001
	Erythromycin	0.04 (− 0.2 to 0.2), 0.68	0.5 (0.2 to 0.7), < 0.001	− 0.4 (− 0.8 to − 0.1), 0.01
Systemic vascular resistance index (SVRI)				
Treatment group	Control	Reference	Reference	Reference
	Methylprednisolone	158 (− 118 to 434), 0.28	− 37 (− 514 to 440), 0.88	320 (49 to 591), 0.038
	Erythromycin	− 54 (− 331 to 224), 0.71	5 (− 473 to 482), 0.98	− 145 (− 422 to 131), 0.33
Time, hours		− 31 (− 36 to − 25), < 0.001	− 19 (− 26 to − 11), < 0.001	− 43 (− 50 to − 36), < 0.001
Interaction between treatment group and time	Control	Reference	Reference	Reference
	Methylprednisolone	15 (9 to 22), < 0.001	11 (2 to 21), 0.023	20 (11 to 28), < 0.001
	Erythromycin	3 (− 4 to 10), 0.38	− 9 (− 19 to 1), 0.06	16 (6 to 25), 0.002
Vasoactive dependency index (VDI)				
Treatment group	Control	Reference	Reference	Reference
	Methylprednisolone	− 0.7 (− 1.7 to 0.3), 0.20	− 1.3 (− 2.6 to 0.06), 0.098	− 0.005 (− 1.5 to 1.4), 0.99
	Erythromycin	− 0.1 (− 1.1 to 0.9), 0.86	− 0.9 (− 2.2 to 0.43), 0.23	0.8 (− 0.6 to 2.3), 0.3
Time, hours		0.07 (0.06 to 0.08), < 0.001	0.02 (0.008 to 0.03), < 0.001	0.1 (0.1 to 0.1), < 0.001
Interaction between treatment group and time	Control	Reference	Reference	Reference
	Methylprednisolone	− 0.06 (− 0.08 to − 0.06), < 0.001	− 0.02 (− 0.03 to − 0.004), < 0.001	− 0.1 (− 0.1 to − 0.1), < 0.001
	Erythromycin	− 0.03 (− 0.03 to − 0.02), < 0.001	0.03 (0.02 to 0.04), < 0.001	− 0.09 (− 0.1 to − 0.07), < 0.001
Lactate (mmol/L)				
Treatment group	Control	Reference	Reference	Reference
	Methylprednisolone	− 0.6 (− 2.1 to 0.9), 0.43	− 1.4 (− 3.7 to 0.9), 0.28	0.2 (− 1.4 to 1.9), 0.80
	Erythromycin	0.4 (− 1.0 to 1.8), 0.6	− 0.4 (− 2.8 to 1.9), 0.73	1.2 (− 0.5 to 2.8), 0.21
Time, hours		0.03 (0.009 to 0.04), 0.002	0.003 (− 0.03 to 0.03), 0.83	0.05 (0.04 to 0.07), < 0.001
Interaction between treatment group and time	Control	Reference	Reference	Reference
	Methylprednisolone	− 0.04 (− 0.06 to − 0.02), < 0.001	− 0.01 (− 0.05 to 0.01), 0.47	− 0.06 (− 0.08 to − 0.04), < 0.001
	Erythromycin	− 0.006 (− 0.03 to 0.02), 0.59	0.01 (− 0.02 to 0.05), 0.44	− 0.02 (− 0.04 to − 0.004), 0.023

Abbreviations: IA: interaction; ID: individual; SD: standard deviation; CI: confidence interval; bpm: beats per minute.

### Secondary outcomes

Animals treated with methylprednisolone displayed a lower shunt fraction, dead space ventilation (Vd/Vt) and plateau pressure (Pplat) (all p for IA pred:time < 0.05) than animals treated with erythromycin or controls which showed similar values over time (Fig. 3A, Table 1). The differences remained significant in OA-LPS treated with methylprednisolone for Vd/Vt, Pplat and additionally EVLWI (all p for IA pred:time < 0.05) while erythromycin was shown a trend towards worse values that did not reach statistical significance except for Pplat (Fig. 3B; Table 1). In OA, parameters were largely comparable among treatment groups and controls except for a significantly lower Pplat and shunt fraction in OA-pred (Fig. 3C, Table 1) There was no significant difference among injury model alone when assessed independently of treatment (ctr, pred and ery combined) (Supplemental Fig. S3, Supplemental Table S2).

#### Hemodynamic status

**Figure 4:**
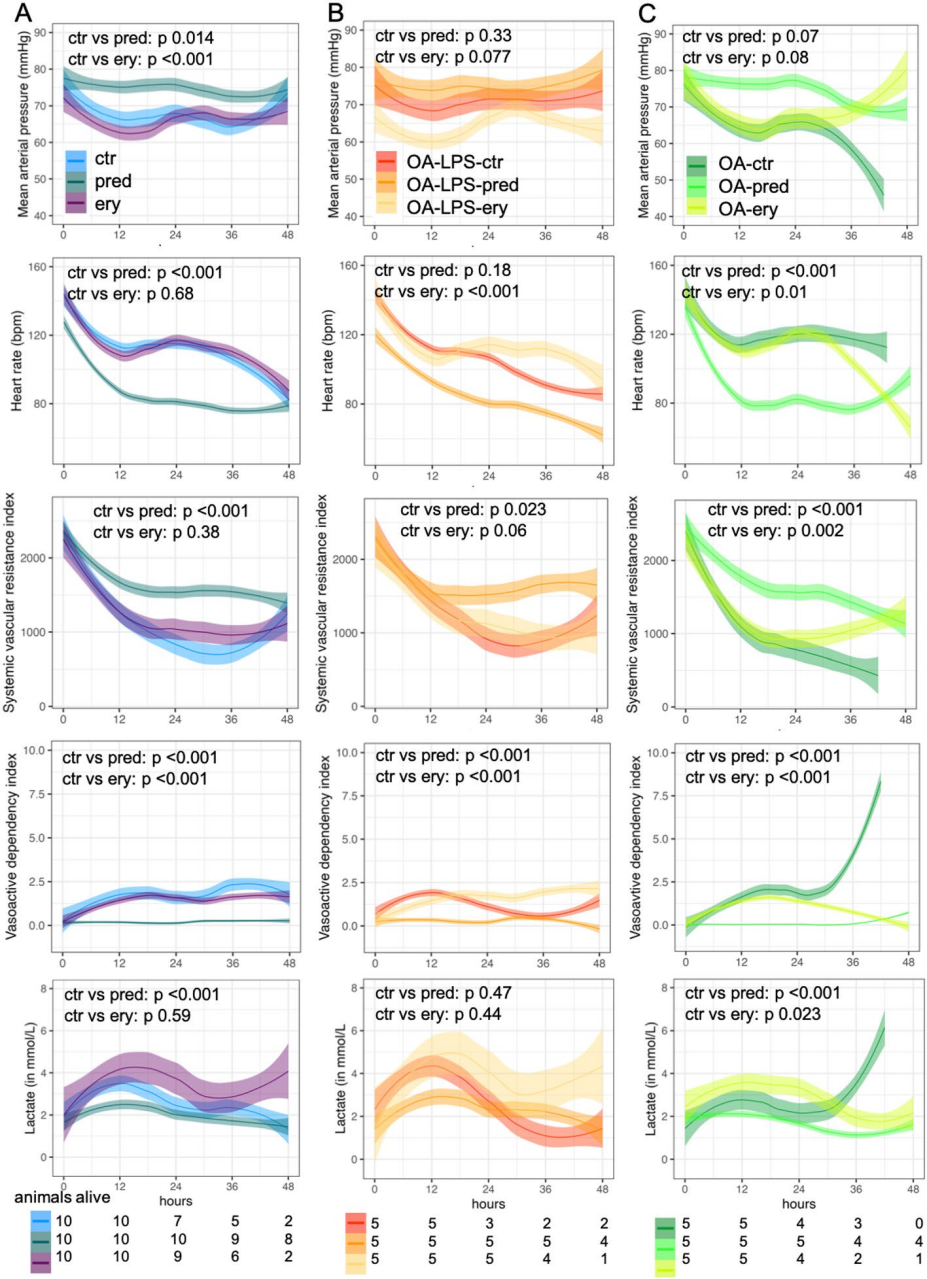
Hemodynamic parameters among (**A**) treatment groups (injury types combined), (**B**) treatment among OA-LPS and (**C**) treatment among OA; displayed in mean over time of observation. Curve smoothened by using the LOESS method (locally estimated scatterplot smoothing), p-values for interaction of treatment group over time. Abbreviations: ctr: control; ery: erythromycin; OA: Oleic Acid; OA-LPS: Oleic Acid and lipopolysaccharides intravenously; pred: methylprednisolone.

MAP, heart rate, SVRI, VDI and lactate clearance displayed better values in animals treated with methylprednisolone when compared to controls (p for IA pred:time < 0.05) while these parameters were shown worse values over time in animals treated with erythromycin as compared to controls (Fig. 4A, Table 1). In both, OA-LPS and OA, treatment with methylprednisolone resulted in a significantly lower VDI and a higher SVRI (p for IA pred:time < 0.05) as compared to controls, while only in OA, HR and lactate displayed statistically significant better values while treated with methylprednisolone as compared to controls (p for IA pred:time < 0.05) (Fig. 4, Table 1). Among injury types, independently of treatment, no difference was observed for all parameters displayed (Supplemental Fig. S3 and Supplemental Table S2).

#### Pulmonary mechanics, fluid balance and metabolic situation

A lower total fluid balance, a higher urinary output with an increased BE was seen in animals treated with methylprednisolone as compared to controls and erythromycin (Supplemental Fig. S5, Supplemental Table S4). Treatment with methylprednisolone facilitated a lower minute ventilation (MV) at a higher compliance and resulted in lower PaCO_2_ values (Supplemental Fig. S6, Supplemental Table S3). No difference was observed among injury models independently of treatment groups (Supplemental Fig. S3 and Supplemental Table S2).

Results of full blood count and biochemistry are shown in Supplemental Fig. S7.

#### Wet-dry ratio and lung injury score

**Figure 5:**
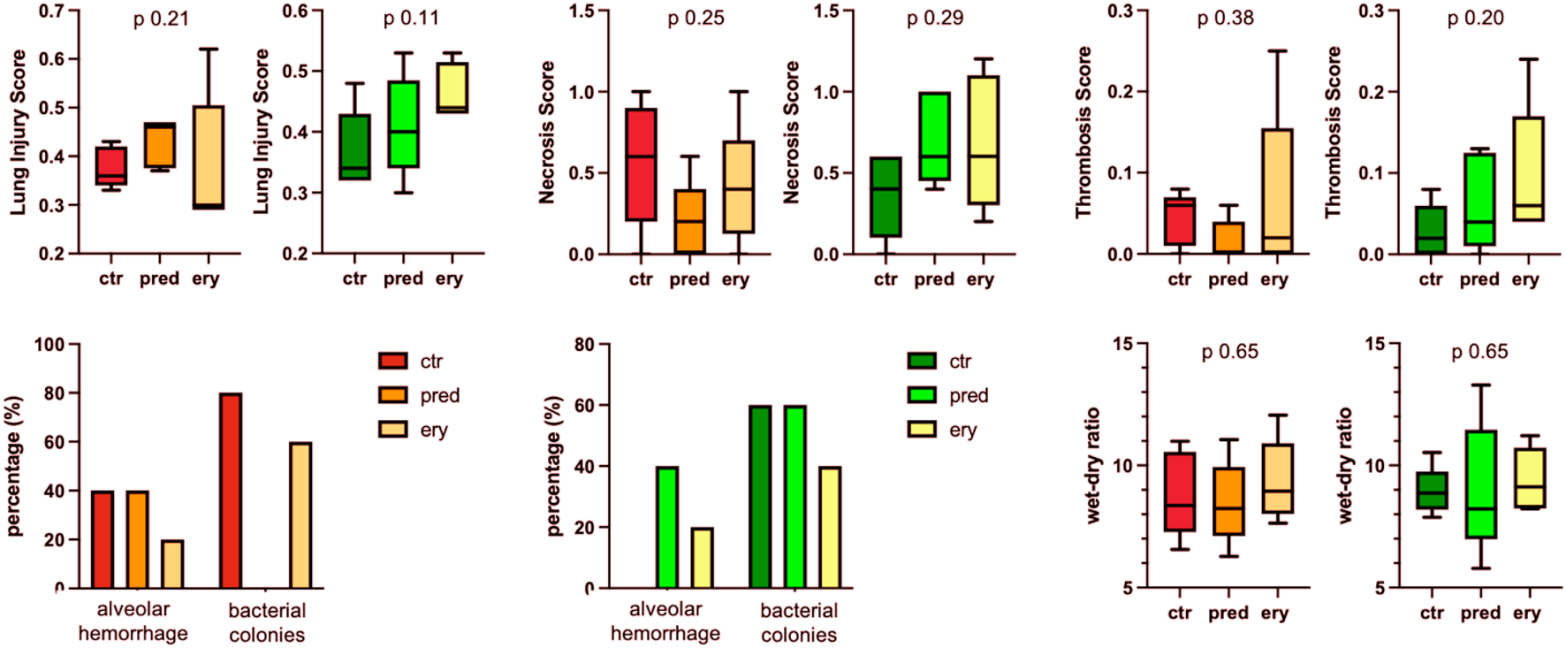
Histopathology assessment of the lungs. In red–orange color palette: OA-LPS (Oleic Acid and lipopolysaccharide) injury model; in green color palette: OA (Oleic Acid) injury model; Whiskers represent 95% confidence interval. Abbreviations: ctr: control; ery: erythromycin; pred: methylprednisolone.

There was no systematic difference among injury models and their respective treatment groups in median wet-dry ratio of the lungs (Fig. 5).

All animals displayed severely damaged lungs in histology assessment. Among OA, the highest LIS was observed in animals treated with erythromycin and the necrosis score was highest among all treated animals (Fig. 5). Among OA-LPS, the LIS, necrosis and thrombosis score was comparable among treatment groups. A higher percentage of bacterial colonies was seen in OA-ctr and -pred as well as OA-LPS-ctr and -ery than in all other groups (Fig. 5).

#### Cumulative survival up to 48 h

Crude survival rates for every animal among treatment groups are shown in Supplemental Fig. S4. Animals treated with methylprednisolone were shown a survival benefit at 48 h (log rank 0.02) (Supplemental Fig. S8A). Among OA-LPS but not OA, control animals displayed a significantly lower survival at 24 h than treated ones (log rank 0.024) (Supplemental Fig. S8B and C.

Deceased animals displayed no difference in the LIS but a trend towards higher necrosis score and more bacterial colonies in histology assessment of the lungs (Supplemental Fig. S9).

#### Predictors of survival and treatment effect

In the Bayesian model, the strongest predictors of survival were compliance, MAP, BE and PF ratio, in ctr vs ery as well as in ctr vs pred in OA-LPS and OA. The strongest predictor for death was lactate, Vd/Vt, mPAP, ventilatory ratio and Pdriv (Supplemental Fig. S10A). Analyzing the treatment effect showed that while pred improved most parameters in comparison with ctr, comparison between ctr and ery did not confirm any benefit associated with the use of erythromycin, specifically in OA animals (Supplemental Fig. S10B, Supplemental Table S4).

## Discussion

This is an evaluation of anti-inflammatory treatment in two different ovine ARDS injury models—OA-LPS, mimicking features of human hyperinflammatory ARDS, and OA alone, as the opposite model—aimed to improve the early course of the disease. We have shown that only in hyperinflammatory ovine ARDS (OA-LPS), treatment with low-dose corticosteroids improved oxygenation significantly as compared to erythromycin and controls. In addition, all animals treated with methylprednisolone, independently of the lung injury type, had a survival benefit up to 48 h, while erythromycin was associated with increased mortality.

Strengths of this study include two different lung injury models with a different inflammatory response but similar clinical presentation, a randomized controlled study design comparing the treatment effect to placebo using an adequate sample size, allocation concealment and blinding. Additionally, an observation period up to 48 h in an ICU setting with rigorous continuous monitoring and the assessment of a variety of clinical parameters (pulmonary mechanics, lung edema, hemodynamics, metabolic situation, fluid balance) are to name.

First and foremost, an early and consistent improvement in oxygenation, as reflected by PF ratio and OI was only shown in the OA-LPS-pred group. A possible pathophysiological explanation for this result is the significantly lower amount of pulmonary edema (as quantified by EVLWI) and dead space ventilation early on after starting treatment. As there was no significant difference in the amount of pulmonary edema (EVLWI and wet-dry ratio) at study end, subsidence of the methylprednisolone effect over time or an additional injury like ventilation-induced damage might be held accountable. Additionally, lower plateau pressures, higher compliance and lower PaCO_2_ were displayed in both injury types treated with methylprednisolone, indicating less pulmonary derangement. These observations are likely related to the specific anti-inflammatory effects of methylprednisolone: amongst corticosteroids as the most potent anti-inflammatory substances, methylprednisolone was chosen for its larger volume of distribution and longer mean residence time, as well as a greater retention in the epithelial lining fluid of the alveoli^25^.

Mechanistically, the pleiotropic effects of corticosteroids are driven by the activation of the glucocorticoid-receptor (GCR) that enables multiple signaling pathways and affects genomic and non-genomic mechanisms^26^. In particular, the proinflammatory pathway via nuclear factor-κB (NF-κB) plays a crucial role in the dysregulation of systemic and pulmonary inflammation in ARDS^27,28^. The GCR inhibits the NF-κB signal pathway^26^ and activator protein-1, inhibiting NF-κB dependent proinflammatory gene expression and the transcription of proinflammatory cytokines^29^. Since LPS but not OA, is known to activate NF-κB^30,31^, part of the beneficial effects of methylprednisolone in our OA-LPS ARDS model might be explained by the inhibition of the NF-κB signalling pathway through the GCR. However, given that corticosteroids interact with the inflammatory cascade on several levels, this mechanism does not explain all observed improvements. In septic shock, corticosteroids reduce levels of pro-inflammatory cytokines IL-6 and -8^32^. In ARDS associated with septic shock, IL-6 decreases over time in treated patients with relative adrenal insufficiency^33^. In our study, we observed the highest plasma IL-6 levels in OA-LPS animals, with levels already decreasing six hours into the study in OA-LPS-pred and OA-LPS-ery, potentially indicating a higher anti-inflammatory effect. There was however no systematic difference in cytokine levels among treatment groups over the whole observation time. Possible explanations are that the treatment benefit regarding oxygenation and hemodynamics is not captured by an effect of pred on cytokine levels or that the granularity of data points with cytokine measurements every 6 h is not matching.

Methylprednisolone was associated with improved hemodynamic stability in comparison with controls or erythromycin. This persisted in OA-LPS-pred and OA-pred, which were both characterized by higher blood pressure and systemic vascular resistance, as well as a lower heart rate and vasoactive need than all other groups. Paired with a lower total fluid balance and a higher base excess in OA-LPS-/OA-pred animals, this indicates a more stable metabolic milieu. The positive effect of methylprednisolone on the hemodynamic parameters could be due to a reversion of a relative or complete adrenal insufficiency in OA-LPS and OA. An indicator for this mechanism is the higher urinary output in OA-LPS-pred and OA-pred, as glucocorticoids suppress the secretion of vasopressin via the neuroendocrinological hypothalamic-pituitary axis^34^. Considering the known effect of corticosteroids on hemodynamic parameters in septic shock^32^, our findings are in line with previous ones.

It is highly likely that the improved hemodynamic and metabolic stability contributed to the improved survival with methylprednisolone throughout the 48 h assessment period in OA-LPS-pred and OA-pred. In the Bayesian model, major differences were observed in treatment effects: comparing controls and methylprednisolone, treatment was shown beneficial for most parameters (in OA-LPS and OA equally), whereas in the comparison of controls and erythromycin, the treatment effect was negative for survival in most parameters, even more so in OA-LPS animals. Yet, our study was likely underpowered to identify any difference in survival between OA and OA-LPS during corticosteroid treatment.

Erythromycin was tested as a second treatment option as it has proven beneficial in chronic inflammatory lung diseases such as chronic obstructive pulmonary disease and bronchiectasis^35,36^ as well as ARDS^37–39^ because of its immunomodulatory effects. Macrolides exhibit multifactorial anti-inflammatory mechanisms by inhibiting chemotaxis, infiltration and activation of neutrophils as well as the production of inflammatory cytokines. In our study, treatment with erythromycin did not provide benefits in any study groups but was associated with a higher risk of death as compared to controls. There are several possible explanations for these results. First, the applied dose, while consistent with previous studies^39,40^, might have been insufficient in the settings of our OA model characterized by life-threating pulmonary inflammation. Second, the 48 h assessment period may have been too short to detect erythromycin-related immunomodulatory effects. Finally, characterization of ARDS subgroups is highly complex and likely not only confined to the inflammatory status, in this context macrolides might have not influenced critical biological pathways. As our understanding of the pathophysiological pathways leading to human hypo- and hyperinflammatory subphenotypes is still imperfect, the likely complex intertwining of general and subphenotypic specific effects cannot be untangled.

Histology overall assessment did not explain the positive treatment response in terms of oxygenation as all animals displayed severe lung injury (LIS) without a systematic difference among type of lung injury or applied treatment. Set in relation with mortality, surviving animals were shown a trend towards lower necrosis score and fewer bacterial colonies in the lung. This remained true for the OA-LPS-pred but not the OA-pred, the two groups with the highest survival rate up to 48 h.

Crucial questions in human ARDS subphenotyping remain to be answered before the translatability of animal models can be fully understood: (A) Is inflammation activation the true differentiating factor among ARDS subphenotypes or is it another yet undiscovered underlying biological process? The answer will determine which treatable traits are the most promising as potential treatment targets. (B) Is the host-response, as measured by clinical parameters and biomarkers in circulation, truly reflecting the biological process in the lung compartment^41^ and in ARDS? Or is it rather an expression of disease severity than specific pathophysiological processes in ARDS, therefore potentially also transmittable to other critical care diseases^42,43^? The immunological processes happening in ARDS might be confined to the lungs, therefore true subphenotypes and possibly ARDS endotypes might only be identified by studying the local compartment.

The present study has several limitations to be addressed: First, the observation period was limited to 48 h, therefore we cannot predict the further course of the assessed parameters. Some clinical and laboratory markers displayed a clear trend and may be even more accentuated in a longer observation period. Second, the limited availability of species-specific research reagents restricts the measurement of several biomarkers as described in the human ARDS population. Third, due to a limited sample size per group, the results regarding survival benefits are likely underpowered. Fourth, blood cultures or other microbiological sampling were not performed, hence we do not know if these animals developed infections, potentially as a side effect of corticosteroids, that might have impacted the inflammatory status or treatment effect.

Due to its pathophysiological complexities, animal models have been crucial to ARDS research: key concepts in ARDS^44^ were originally discovered in large animal models before successful application in human^2^. As preclinical models can not fully reproduce human ARDS in all its complexity, by narrowing the complexity of the disease to more homogenous subgroups, the chances of identification of treatable traits increase^45^. Likely this is the gap where translational models could be valuable: to reproduce a specific treatable trait and test targeted treatment. Our ovine model offers opportunities to investigate OA-LPS, which resembles in many features the human hyperinflammatory subphenotype^16^ as similar pathways of inflammation are activated^15,17^. A comparison of omics data among preclinical models and human ARDS subphenotypes could inform about the true extent of comparability.

## Conclusion

Early and persistent improvement in oxygenation was seen only in hyperinflammatory ovine ARDS treated with methylprednisolone, while hemodynamic situation and survival was improved in both injury models and corticosteroid treatment. Erythromycin did not offer any benefit regarding all assessed outcomes.

### Supplementary Information

Supplementary Information.

## Data Availability

The dataset generated and analysed during the current study are not publicly available due to being part of a PhD project but are available from the corresponding author on reasonable request.

## Abbreviations

ARDS: Acute Respiratory Distress Syndrome
ATS: American Thoracic Society
BAL: Bronchoalveolar lavage
BE: Base excess
CI: Confidence interval
CVL: Central venous line
ELISA: Enzyme-linked immunoassay
EtCO_2_: End-tidal carbon dioxide
EVLWI: Extravascular lung water index
FiO_2_: Inspired oxygen fraction
GCR: Glucocorticoid receptor
GEDI: Global end-diastolic index
HR: Heart rate
IFNγ: Interferon gamma
IL: Interleukin
IQR: Interquartile range
IV: Intravenously
LIS: Lung injury score
LMM: Linear-mixed effects models
LPS: Lipopolysaccharide
MAP: Mean arterial pressure
mPAP: Mean pulmonary artery pressure
NS: Normal saline
OA: Oleic acid
OA-ctr: Oleic acid control
OA-pred: Oleic acid treated with methylprednisolone
OA-ery: Oleic acid treated with erythromycin
OA-LPS: Oleic acid and LPS intravenously
OA-LPS-ctr: Oleic acid and LPS intravenously control
OA-LPS-pred: Oleic acid and LPS intravenously treated with methylprednisolone
OA-LPS-ery: Oleic acid and LPS intravenously treated with erythromycin
OI: Oxygenation index
Pdriv: Driving pressure
PF ratio: PaO_2_/FiO_2_ ratio
PEEP: Positive end-expiratory pressure
Pplat: Plateau pressure
PiCCO: Pulse contour cardiac output
PPV: Pulse pressure variation
QUT: Queensland University of Technology
SD: Standard deviation
SpO_2_: Oxygen saturation
SVV: Stroke volume variation
Vd/Vt: Dead space ventilation
