# Supplementary material for: Application of anti-inflammatory treatment in two different ovine Acute Respiratory Distress Syndrome injury models: a preclinical randomized intervention study
Source: Sci Rep. 2023 Oct 20;13:17986. doi: 10.1038/s41598-023-45081-8 (PMC10589361; doi:10.1038/s41598-023-45081-8)
Supplement: Supplementary file 1 — Supplementary Information. [file 41598_2023_45081_MOESM1_ESM.docx]

**ONLINE SUPPLEMENT: Application of anti-inflammatory treatment in two different ovine ARDS injury models – a preclinical randomized intervention study**

**Supplemental methods**

1. ARRIVE guidelines: checklist
2. Intra-experimental monitoring and management: Supplemental Figure S1 and Supplemental Figure S2
3. Data recording throughout the experiment
4. General sampling and inflammatory cytokines, histopathology
5. Formulas used for calculation of oxygenation index, dead space ventilation, shunt fraction and vasoactive dependency index
6. Minimal reproducible R code a) for the linear mixed effect model b) for the figures (line graphs over time with LOESS smoothing) and c) for the Bayesian joint model for survival

**Supplemental results**

1. Baseline characteristics: Supplemental Table S1
2. Pairwise comparison over time among injury type (treatment groups and control

combined): Supplemental Figure S3 and Supplemental Table S2

1. Crude survival times in main study: Supplemental Figure S4
2. Fluid balance and additional hemodynamic parameters: Supplemental Figure S5
3. Additional respiratory mechanics parameters: Supplemental Figure S6
4. Linear mixed-effect model for respiratory mechanics and additional fluid and hemodynamic parameters: Supplemental Table S3
5. Results of full blood count and biochemistry: Supplemental Figure S7
6. Cumulative survival among A) treatment groups (injury types combined) B) treatment among OA-IV-LPS and C) treatment among OA: Supplemental Figure S8
7. Lung Injury Score among deceased and surviving animals: Supplemental Figure S9
8. Bayesian model for predictors of survival and treatment effect: Supplemental Figure S10
9. Model specification and detailed results of Bayesian joint model for survival and treatment effect: Supplemental Table S4

**Supplemental methods**

**1) ARRIVE guidelines: checklist** (1)

|  | | ITEM | RECOMMENDATION | Section/ Paragraph |
| --- | --- | --- | --- | --- |
| 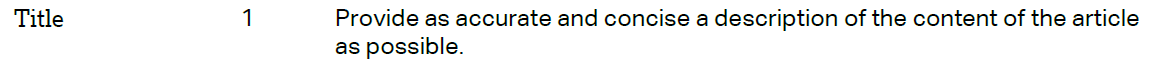 | | | Page 1 |  |
| 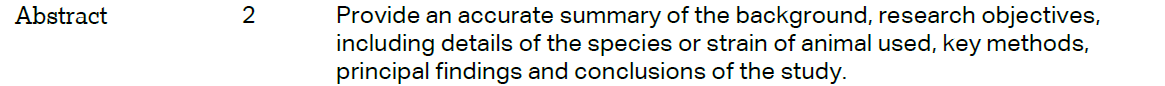 | | | Page 2 |  |
| INTRODUCTION | | |  |  |
| 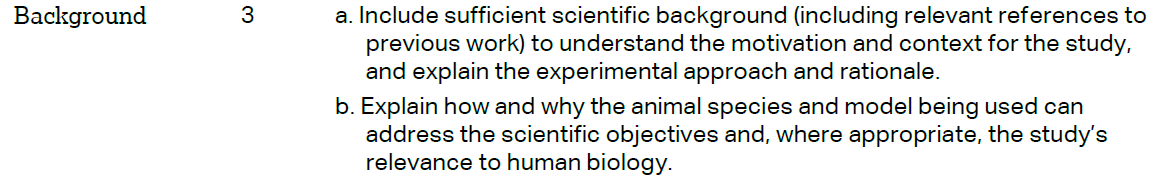 | | | Page 5-6 |  |
| 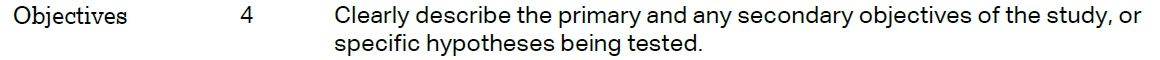 | | | Page 6 |  |
| METHODS | | |  |  |
| 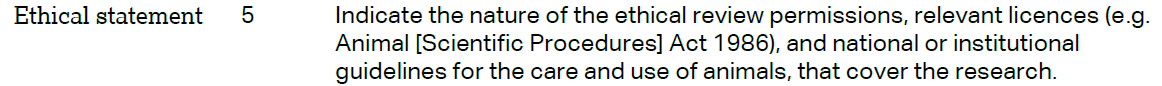 | | | Page 7 |  |
| 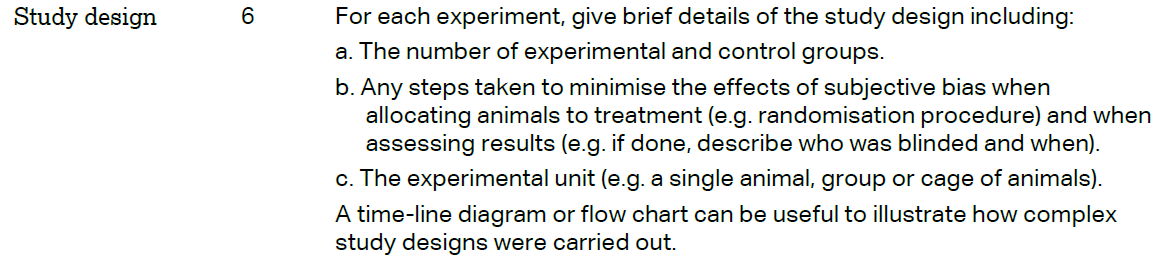 | | | Page 7  Page 7/9,  Fig 1  Page 7  Fig 1 |  |
| 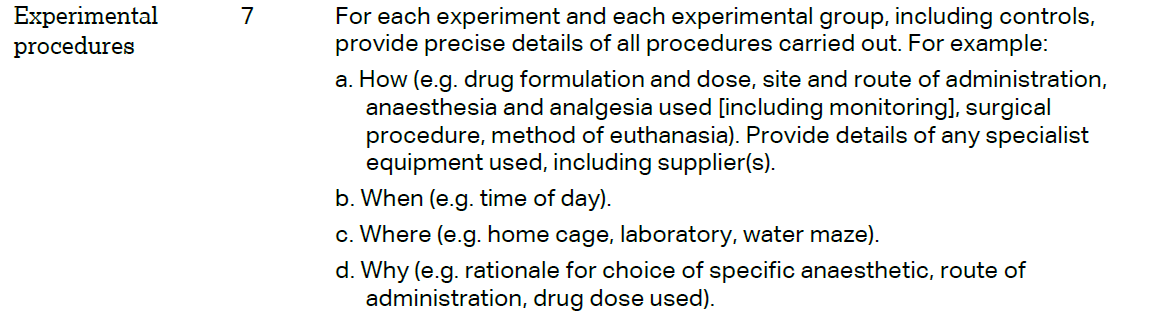 | | | Page 7-8  OS, page 4-6  Page 7,19/20 |  |
| 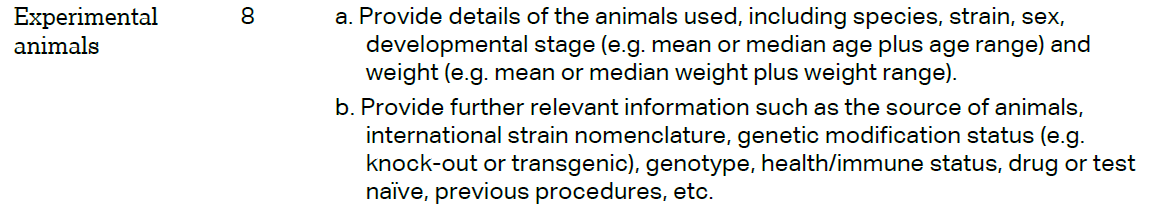 | | | Page 13,  eTable S1 |  |
| 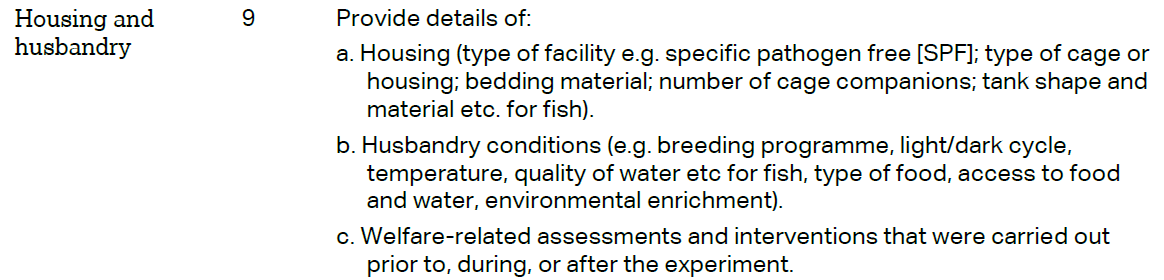 | | | NA  page 7 |  |
| 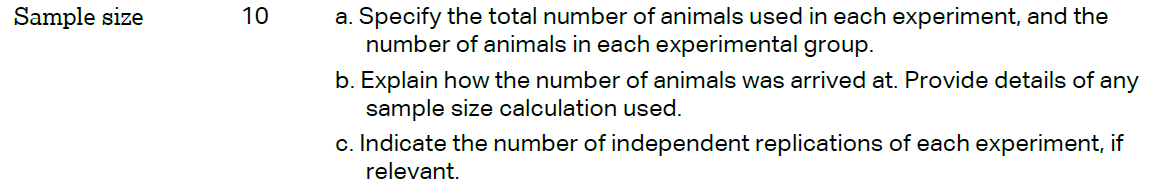 | | | Page 7, Fig 1  Page 11-12  Page 7 |  |
| 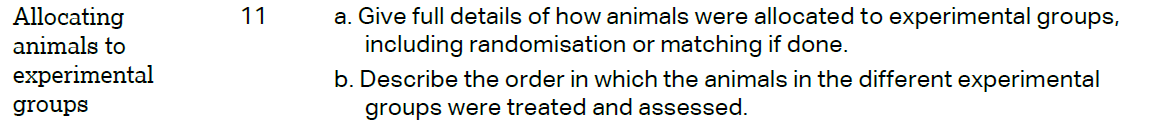 | | | Page 7 |  |
| 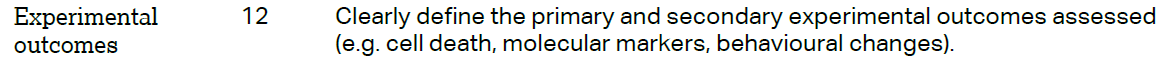 | | | Page 10 |  |
| 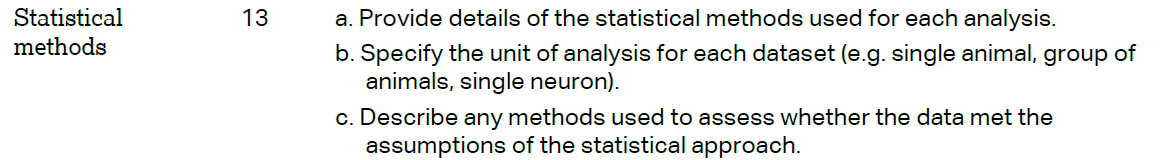 | | | Page 11-12  Page 7  Page 7 |  |
| **RESULTS** | | |  |  |
| 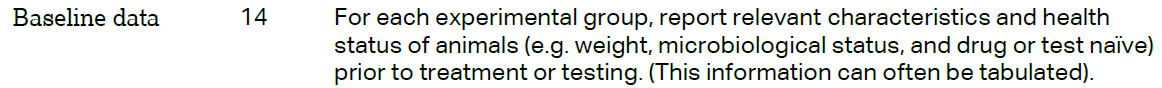 | | | OS, eTable S1 |  |
| 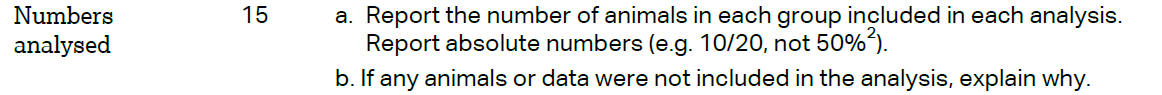 | | | Page 13, Fig 1 |  |
| 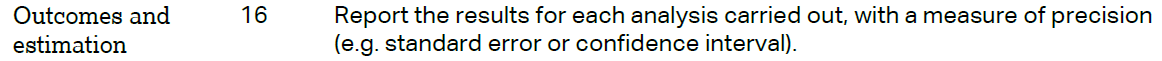 | | | Page 13-16; Figure 1-5; Table 1 |  |
| 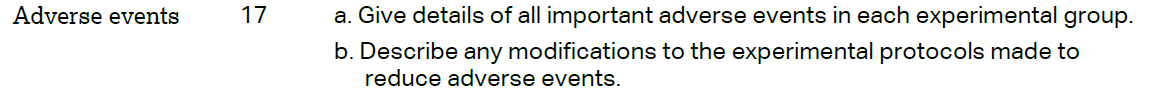 | | | NA |  |
| **DISCUSSION** | | |  |  |
| 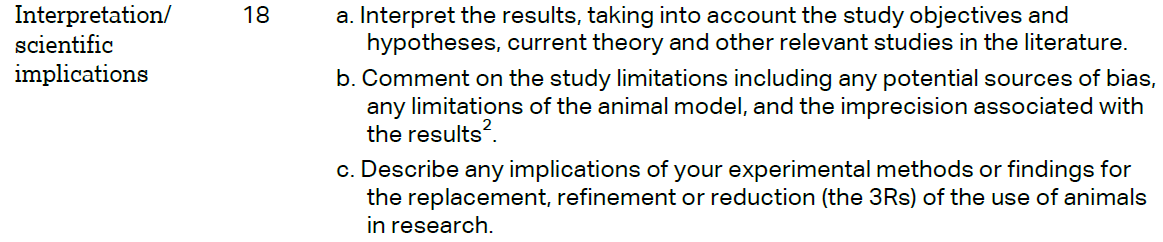 | | | Page 17-21  Page 20  Page 19/20 |  |
| 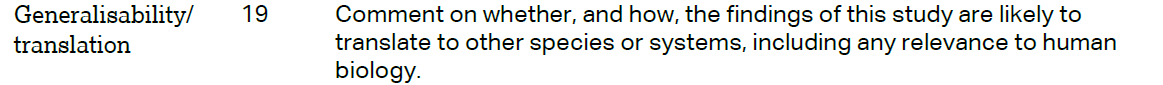 | | | Page 20-21 |  |
| 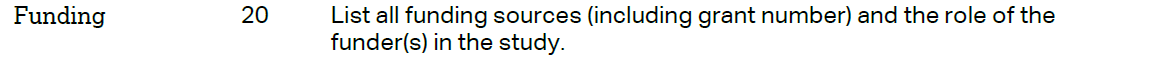 | | | Page 25-26 |  |

**2) Intra-experimental monitoring and management: Supplemental Figure S1 and Figure S2**

Normothermia was maintained via external warming. Parameters of pulmonary mechanics and hemodynamics, drug consumption and fluid balance were recorded hourly. Protocolized administration of vasoactive drugs was used if a mean arterial pressure of ≥ 65mmHg could not be maintained and the PiCCO parameters were not indicative of fluid responsiveness (SVV and PPV<12%, GEDI >900). Noradrenaline 0.01-0.3µg/kg/min IV was used as first-line agent, in face of refractory hypotension, metaraminol 1-5mg/h and vasopressin 0.01-0.1 U/min IV were added (**Supplemental Figure S1**).

After confirmation of ARDS, mechanical ventilation settings were adjusted as according to the EXPRESS trial (2): tidal volume of 6ml/kg, FiO_2_ to maintain an arterial blood gases SpO_2_ of >88% and PEEP to maintain a plateau pressure (Pplat) <30cmH_2_O (**Supplemental Figure S2**).

**Supplemental Figure S1:** strategy for vasoactive/hemodynamic and fluid management

**
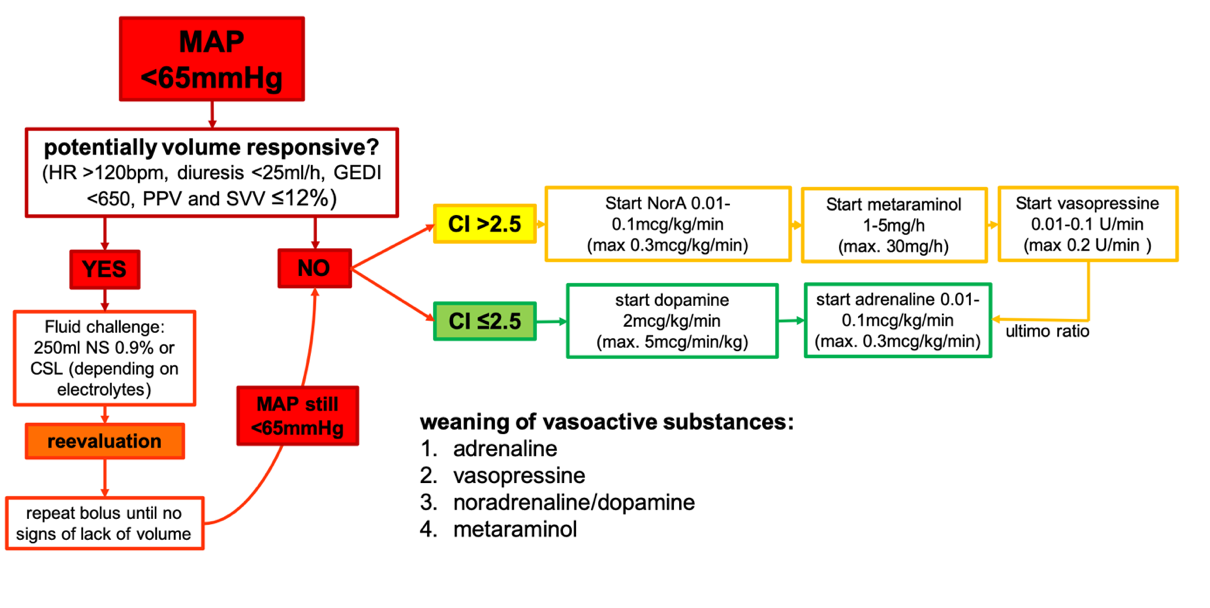
**

Abbreviations: bpm = beats per minute; CI = cardiac index; CSL = Hartmanns solution; GEDI = Global end-diastolic index; HR = heart rate; MAP = mean arterial pressure; mcg = microgram; min = minute; NorA = noradrenaline; NS = normal saline; PPV = pulse pressure variation; SVV = stroke volume variation

**Supplemental Figure S2:** Strategy for mechanical ventilation


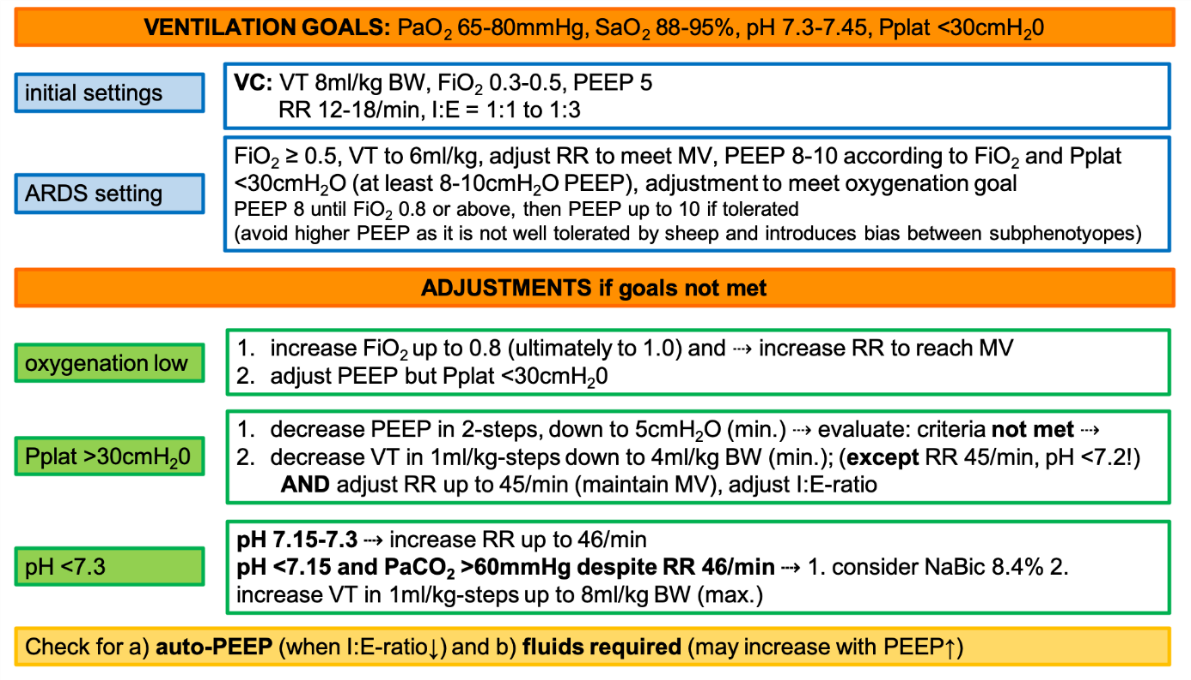


Abbreviations: BW = body weight; FiO_2_ = inspired oxygen fraction; I:E = inspiration-to-expiration time; MV = minute ventilation; PaO_2_ = arterial oxygen partial pressure; PEEP = positive end-expiratory pressure; Pplat = plateau pressure; RR = respiratory rate; SaO_2_ = arterial oxygen content; VC = volume controlled ventilation mode; VT = tidal volume

**3) Data recording throughout the experiment**

Hemodynamic parameters were captured at 1 kHz on a 16-channel PowerLab data acquisition system (model ML880) recorded with Labchart 7 (AD Instruments, Bella Vista, Australia). Invasive arterial blood pressure, central venous pressure, pulmonary artery pressure, ECG, heart rate, SpO_2_ and EtCO_2_ were continuously monitored (Marquette Solar 8000, GE Healthcare, Chicago, ILL, USA) throughout the experiment. Continuous cardiac output, preload parameters (stroke volume variation, pulse pressure variation and global enddiastolic index) and systemic vascular resistance index were continuously measured with the PiCCO system (PULSION Medical Systems, Getinge, Gothenburg, Sweden). Transpulmonary thermodilution to calibrate the system and assess extravascular lung water index was performed every two hours or more often if deemed necessary to assess the clinical status of the animal and manage fluids and vasoactive medication (as outlined above). Body surface area (BSA) was calculated using the following equation, BSA = 0.094 × (body weight in kg)^0.67^ (3). Mixed venous oxygen saturation (SvO_2_), pulmonary artery pressures and core temperature was monitored using Vigilance II Monitor (Edward Lifesciences, Irvine, CA, USA). Custom software by the manufacturer was used to record ventilatory parameters of the Hamilton-G5 ventilator at 100 Hz. Animals were continuously monitored by trained clinical staff throughout the course of the study. Fluid balance was checked hourly and blood gases every 2 hours or more often if clinically necessary for the management of the animal. The results were used to manage the animals accordingly (as outlined above). In addition to the automated data acquisition system, parameters of hemodynamics, mechanical ventilation and pulmonary mechanics as well as fluids were recorded at baseline, and then every hour thereafter until study end using a tablet (iPad, Apple, Cupertino) connected to an online database with automatic recording.

**4) General sampling and for inflammatory cytokines, histopathology**

At each predefined point (**Figure 1B**: Tb, T-1, T2, T6, T12, T18, T24, T30, T36, T42 and T48) arterial blood samples were taken as 4x EDTA tubes (4 ml), 3x citrate tubes (3.5 ml), 2x serum tubes (5 ml) and 1x lithium heparin (6 mL), resulting in a total of 42.5 ml drawn blood volume. At Tb, T12, T24, T36 and T48, an additional 8.5 ml (1x citrate and serum tube) was taken for FBC, biochemistry and coagulation tests. Additionally, blood gas analysis was performed every 2 hours and whenever deemed clinically necessary, resulting in a drawn blood volume of 0.5ml each time.

Positive internal controls of inflammatory cytokines were used to ensure that inter- and intra-plate variability was <10% and to confirm the precision of all the assays. The standard curve ranges were 312.5 – 20000 pg/mL for IL-6, 31.25 – 2000 pg/mL for IL-8, 125 – 8000 pg/mL for IL-10 and 46.8 – 3000 pg/mL for IL-1β (4). Interferon gamma (IFNy) was quantified using a custom Luminex immunoassay (Milliplex Ovine Cytokine/Chemokine Panel 1; Merck Millipore, USA) in accordance with the manufacturers instructions. Concentrations were calculated using a 5-parameter logistic standard curve, corrected for background readings. Assay sensitivity (minimum detectable concentration; pg/mL) for IFNy was 0.18.

Lung tissues at study end were preserved in 10% neutral buffered formalin for 24 hours, embedded in paraffin, sectioned to a 5 µm thickness and stained with hematoxylin&eosin using standard procedures. Slides were examined by independent, blinded, qualified veterinary pathologist and lung injury score was assessed as recommended by the ATS for experimental ARDS in animal models (5).

**5) Formulas used for calculation of oxygenation index, dead space ventilation, shunt fraction and vasoactive dependency index**

**Oxygenation index (OI):** dimensionless number; as according to (6)

OI = (FiO_2_ x Pplat) / PaO_2_

FiO_2:_ inspired oxygen fraction; Pplat: plateau pressure; PaO_2_: arterial oxygen partial pressure

**Dead space ventilation (Vd/Vt):** in [%]; as according to Enghoff formula (7)

Vd/Vt = ((PaCO_2_ – EtCO_2_)/PaCO_2_)

PaCO_2_: arterial carbondioxid partial pressure; EtCO_2_: endtidal carbondioxid

**Shunt fraction (Qs/Qt):** in [%]; as according to (8)

(Qs/Qt) = (CcO_2_ – CaO_2_) / (CcO_2_ – CmvO_2_)

CcO_2_: alveolar oxygen content; CaO_2_: arterial oxygen content; CvO_2_: mixed-venous oxygen content

**Vasoactive dependency index (VDI):** dimensionless number; as according to (9)

VDI = (dopamine (in mcg/kg/min) + dobutamine (in mcg/kg/min) + (100 x noradrenaline (in mcg/kg/min)) + (100 x adrenaline (in mcg/kg/min)) + (1000 x vasopressine (in U/kg/min))) / MAP

MAP: mean arterial pressure; U: units

**6) Minimal reproducible R code**

1. **for the linear mixed effect model**

this example: oxygenation parameters among treatment groups in OA-LPS injury model

**Packages used:** “lmerTest”, “tidyverse”, “ggplot2”, “ggeffects”, “emmeans”

stardLongOAIVLPS <- read_excel("~/Library/MobileDocuments/stardLongOAIVLPS.xlsx")

**#convert treatment to a categorical variable (1 = ctl, 2 = pred, 3 = ery)**

stardLongOAIVLPS = stardLongOAIVLPS %>% mutate_at('treatment',~factor(.,levels=1:3,labels=c('ctl','pred','ery')))

**#fit models in order - random intercept only; all models in a single list to summarise output**

model_fits <- list()

## PF ratio ##

model_fits[['PFratio']] = lmer(PFratio ~ treatment*hour + (1 | ID), data=stardLongOAIVLPS)

## OxygIndex ##

model_fits[['OxygIndex']] = lmer(OxygIndex ~ treatment*hour + (1 | ID), data=stardLongOAIVLPS)

## EVLWI ##

model_fits[['EVLWI']] = lmer(EVLWI ~ treatment*hour + (1 | ID), data=stardLongOAIVLPS)

## VD_vent ##

model_fits[['VD_vent_perc']] = lmer(VD_vent ~ treatment*hour + (1 | ID), data=stardLongOAIVLPS)

## Shunt ##

model_fits[['Shunt']] = lmer(Shunt ~ treatment*hour + (1 | ID), data=stardLongOAIVLPS)

**#summarise output - fixed effects estimates and hypothesis testing -> lapply() to loop over all of the models in model_fits**

model_summaries = lapply(seq_along(model_fits),function(x) summary(model_fits[[x]])$coefficients %>% data.frame() %>% rownames_to_column(var='Fixed effect'))

**#assign model names**

names(model_summaries) <- names(model_fits)

**#combine into one table**

model_summaries = bind_rows(model_summaries,.id='Outcome')

**#extract confidence intervals and add to model_summaries**

model_cis = lapply(seq_along(model_fits),function(x) confint(model_fits[[x]]) %>% data.frame() %>% rownames_to_column(var='Fixed effect') %>% filter(grepl('treatment|hour',`Fixed effect`)))

**#assign model names**

names(model_cis) <- names(model_fits)

model_cis = bind_rows(model_cis,.id='Outcome')

**#join to model_summaries & tidy up names**

model_summaries = full_join(model_summaries,model_cis,by=c('Outcome','Fixed effect')) %>% rename('SE'=Std..Error,'p-value'=Pr...t..,'lower CI95'=X2.5..,'upper CI95'=X97.5..)

View(model_summaries)

1. **for the figures (line graphs over time with LOESS smoothing)**

## PF

mean_dataPFratio<-group_by(stardLongOAIVLPS, treatment, hour) %>%

summarise(PFratio=mean(PFratio, na.rm=TRUE))

ggplot(na.omit(mean_dataPFratio), aes(x=hour, y=PFratio, colour=treatment, fill=treatment)) +

geom_line() +

scale_color_manual(values = c('#FF3300','#FF9900', '#fed976')) +

scale_fill_manual(values = c('#FF3300','#FF9900', '#fed976')) +

scale_x_continuous(breaks=seq(0,48,12)) +

stat_smooth(method="loess", size=0.5) +

theme_bw(base_size=18) +

ylim(50, 350) +

ggtitle("") + xlab("hours") + ylab("PaO2/FiO2 ratio") +

ggsave("LG_Ph2_PF48h.png", width = 7, height = 5)

1. **for the Bayesian joint model for survival**

this example: lactate among control and treatment with methylprednisolone in OA-LPS injury model

**Packages used:** “rstanarm” v2.21.1

**##data**

>stardSurvPh2ctrster

>stardLongPh2ctrster

**##Bayesian joint model: univariate**

>modlactate <- stan_jm(formulaLong = lactate ~ trt + hour + (hour | ID),

dataLong = stardLongPh2ctrster,

formulaEvent = survival::Surv(Futimehours, death) ~ trt,

dataEvent = stardSurvPh2ctrster,

time_var = "hour",

basehaz_ops = list(df=4),

chains = 1, refresh = 2000, seed = 12345)

>print(modlactate)

**##hazard ratio for assessed parameter and treatment effect**

>summary(modlactate)

**##get 95% credible interval**

>posterior_interval(modlactate, probs=0.95)

**Supplemental results**

**1) Baseline characteristics: Supplemental Table S1**

|  | All, n=30 | OA, n=15 | OA-LPS, n=15 | p |
| --- | --- | --- | --- | --- |
| Weight (kg) | 51 (47-52) | 52 (47-53) | 49 (47-52) | 0.35 |
| Body surface area (per m^2^) | 1.30 (1.22-1.34) | 1.33 (1.24-1.34) | 1.28 (1.22-1.32) | 0.29 |
| Temperature (°C) | 38.1 (0.7) | 38.2 (0.6) | 38.0 (0.8) | 0.44 |
| Hemodynamic parameters |  |  |  |  |
| Mean arterial blood pressure (mmHg) | 114 (13) | 111 (16) | 117 (10) | 0.16 |
| Heart rate (bpm) | 77 (28) | 75 (29) | 78 (28) | 0.68 |
| Mean pulmonary artery presure (mmHg) | 13 (11-14) | 13 (11-17) | 13 (11-14) | 0.68 |
| Cardiac index | 4.6 (1.5) | 4.5 (1.7) | 4.7 (1.3) | 0.33 |
| Systemic vascular resistance index | 2173 (604) | 2245 (567) | 2101 (650) | 0.46 |
| Global enddiastolic index | 1335 (287) | 1329 (312) | 1341 (270) | 0.94 |
| Mixed-venous oxygenation (%) | 77 (71-82) | 75 (68-81) | 80 (71-85) | 0.14 |
| Mechanical ventilation |  |  |  |  |
| Minute ventilation (L/min) | 6.1 (0.8) | 6.1 (0.9) | 6.1 (0.6) | 0.90 |
| Compliance (mL/cmH_2_O) | 40 (38-44) | 40 (35-44) | 38 (31-41) | 0.25 |
| PEEP (cmH20) | 5 (5-5) | 5 (5-5) | 5 (5-5) | 0.78 |
| Plateau pressure (cm H_2_O) | 14.8 (13.5-15.9) | 14.1 (13.0-15.3) | 15.0 (14.0-16.0) | 0.23 |
| Extravascular lung water index | 25.5 (23.8-30.3) | 25.0 (23.0-30.0) | 26.0 (24.0-31.0 | 0.62 |
| EtCO2 | 43.9 (6.0) | 43.9 (6.8) | 43.9 (5.5) | 0.71 |
| Blood gases |  |  |  |  |
| PaO_2_/FiO_2_ ratio | 514 (469-539) | 514 (472-532) | 514 (460-526) | 0.94 |
| PaCO2 (mmHg) | 40.4 (5.1) | 40.9 (4.8) | 39.9 (5.5) | 0.57 |
| Oxyhemoglobin | 98.3 (98.1-98.7) | 98.4 (98.1-98.8) | 98.3 (97.8-98.6) | 0.31 |
| Lactate | 0.6 (0.4-0.9) | 0.6 (0.5-1.0) | 0.5 (0.4-0.7) | 0.44 |
| Base excess (mmol/L) | 2.3 (2.3) | 2.2 (1.9) | 2.4 (2.7) | 1.0 |
| Bicarbonate (mmol/L) | 26.2 (2.3) | 26.1 (2.0) | 26.2 (2.7) | 1.0 |
| Full blood count |  |  |  |  |
| Hemoglobin (g/L) | 112 (8.6) | 110 (9.4) | 114 (7.5) | 0.33 |
| Platelets (x10^9/L) | 366 (103) | 342 (112) | 390 (91) | 0.12 |
| Neutrophil count (x10^9/L) | 2.0 (0.7) | 2.0 (0.6) | 1.9 (0.9) | 0.33 |
| Lymphocyte count (x10^9/L) | 3.2 (0.7) | 3.2 (0.7) | 3.3 (0.7) | 0.87 |
| Coagulation |  |  |  |  |
| PT (sec) | 14.0 (0.9) | 13.8 (0.5) | 14.2 (1.1) | 0.25 |
| aPTT (sec) | 27.3 (4.0) | 25.9 (2.9) | 28.5 (4.4) | 0.15 |
| Biochemistry |  |  |  |  |
| Sodium (mmol/L) | 143 (2) | 144 (2) | 143 (2) | 0.60 |
| Potassium (mmol/L) | 4.5 (4.3-4.7) | 4.4 (4.3-4.7) | 4.5 (4.4-4.7) | 0.44 |
| Creatinine (mmol/L) | 0.08 (0.06-0.08) | 0.08 (0.07-0.08) | 0.08 (0.06-0.08) | 0.87 |
| Urea (mmol/L) | 7.4 (1.4) | 7.7 (1.4) | 7.0 (1.4) | 0.19 |
| Albumin (g/L) | 35 (2.7) | 35 (3.3) | 35 (1.9) | 0.65 |
| Bilirubin (umol/L) | 3 (3-4) | 3 (3-4) | 4 (3-4) | 0.35 |
| ASAT (IU/L) | 93 (21) | 87 (19) | 99 (22) | 0.25 |
| ALP (IU/L) | 127 (93-207) | 135 (92-206) | 120 (102-214) | 0.78 |
| gGT (IU/L) | 48 (9) | 49 (10) | 47 (7) | 078 |
| GLDH (IU/L) | 13 (9-26) | 12 (8-17) | 14 (9-33) | 0.35 |
| CK (IU/L) | 154 (114-204) | 150 (109-189) | 157 (116-352) | 039 |
| Cytokines |  |  |  |  |
| IL-6 plasma (pg/ml) | 313 (313-313) | 313 (313-313) | 313 (313-313) | 0.54 |
| IL-8 plasma (pg/ml) | 816 (306-1837) | 1433 (586-2022) | 547 (305-1583) | 0.14 |
| IL-10 plasma (pg/ml) | 596 (380-14033) | 750 (513-1073) | 499 (258-1020) | 0.57 |
| IL-1β plasma (pg/ml) | 149 (97-268) | 143 (98-210) | 160 (92-313) | 0.12 |
| IFNy plasma (pg/ml) | 1.6 (0.8-2.1) | 1.5 (0.7-2.0) | 1.7 (1.0-3.0) | 0.49 |
| Dose of Oleic Acid (ml/kg) | 0.14 (0.012-0.18) | 0.17 (0.11-0.19) | 0.13 (0.11-0.17) | 0.64 |

Abbreviations: OA = Oleic Acid; OA-LPS = Oleic Acid and LPS; PEEP = positive end-expiratory pressure; EtCO2 = endtidal carbondioxide; PaCO2 (mmHg) = arterial carbondioxide partial pressure; PT = prothrombin time; aPTT = activated partial thromboplastin time; ASAT = aspartate transaminase; ALP = alkaline phosphatase; gGT = gamma-glutamyl transferase, GLDH = glutamate dehydrogenase; CK = creatin kinase, IL = interleukin; IFNy = interferon gamma

**2) Pairwise comparison over time among injury type (treatment groups and control**

**combined): Supplemental Figure S3 and Supplemental Table S2**

**Supplemental Figure S3:** all oxygenation, hemodynamic, metabolic and pulmonary mechanic parameters among the injury types (all treatment groups combined for each lung injury type)


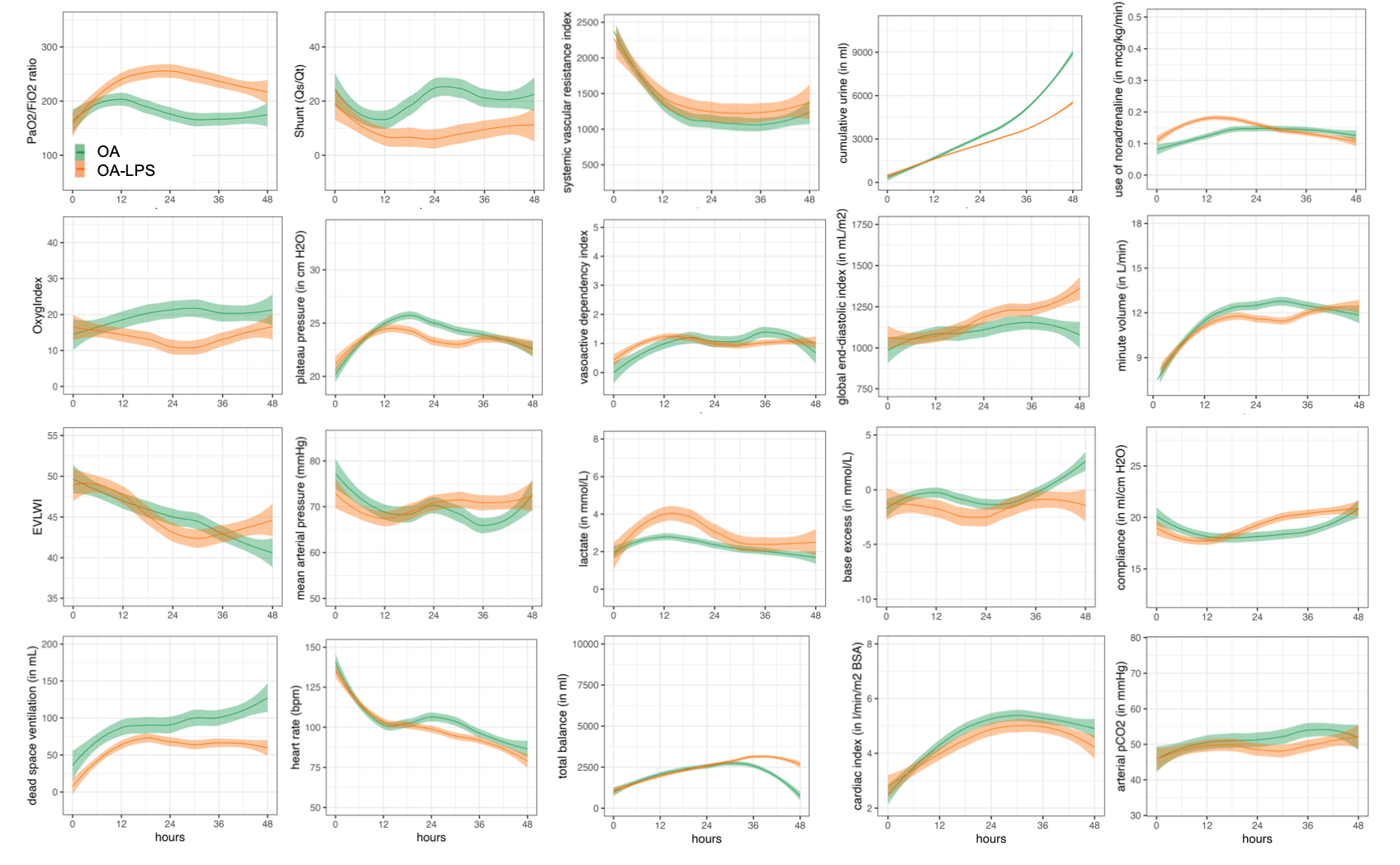


Abbreviations: OA = Oleic Acid; OA-LPS = Oleic Acid and lipopolysaccharides

**Supplemental Table S2:** pairwise comparison over time among injury types (treatment groups combined for each injury type)

| **Outcome** | **Estimate** | **Standard error** | **Degree of freedom** | **p value** |
| --- | --- | --- | --- | --- |
| PaO_2_/FiO_2_ ratio (PF ratio) | -52.1 | 26.4 | 28 | 0.06 |
| Oxygenation Index (OI) | 6.9 | 5.1 | 28 | 0.19 |
| Extravascular lung water index (EVLWI) | 0.9 | 2.6 | 28 | 0.74 |
| Dead space ventilation (%) | 32.7 | 24.9 | 28 | 0.20 |
| Shunt (Qs/Qt) | 10.7 | 5.3 | 28 | 0.05 |
| Plateau pressure (cm H_2_O) | 1.1 | 1.8 | 28 | 0.55 |
| Mean arterial blood pressure (mmHg) | -1.3 | 2.6 | 28 | 0.62 |
| Heart rate (bpm) | 2.7 | 6.7 | 28 | 0.69 |
| Systemic vascular resistance index (SVRI) | -196.1 | 137.3 | 28 | 0.16 |
| Lactate (mmol/L) | -0.7 | 0.6 | 28 | 0.29 |
| Vasoactive depencency index (VDI) | 0.0 | 0.5 | 28 | 0.95 |
| Total balance (ml) | 21.6 | 493.2 | 28 | 0.97 |
| Cumulative urine (ml) | 490.9 | 449.5 | 28 | 0.28 |
| Global end-diastolic index (ml/min/m^2^) | -32.3 | 67.6 | 28 | 0.64 |
| Base excess (mmol/L) | 1.0 | 1.1 | 28 | 0.40 |
| Cardiac index (L/min/m^2^ BSA) | 0.4 | 0.4 | 28 | 0.24 |
| Noradrenaline dose (mcg/kg/min) | 0.0 | 0.0 | 28 | 0.66 |
| Minute ventilation (L/min) | 0.9 | 1.3 | 28 | 0.48 |
| Compliance (ml/cm H_2_O) | -0.8 | 1.4 | 28 | 0.57 |
| Arterial CO_2_ partial pressure (mmHg) | 3.2 | 3.7 | 28 | 0.39 |

Abbreviations: bpm = beats per minute; ml = milliliter, mcg = microgram; BSA = body surface area; L = liter; kg = kilogram; cm = centimeter; CO2 = carbondioxide; mmHg = millimeter mercury

**3) Survival times in main study: Supplemental Figure S4**

**
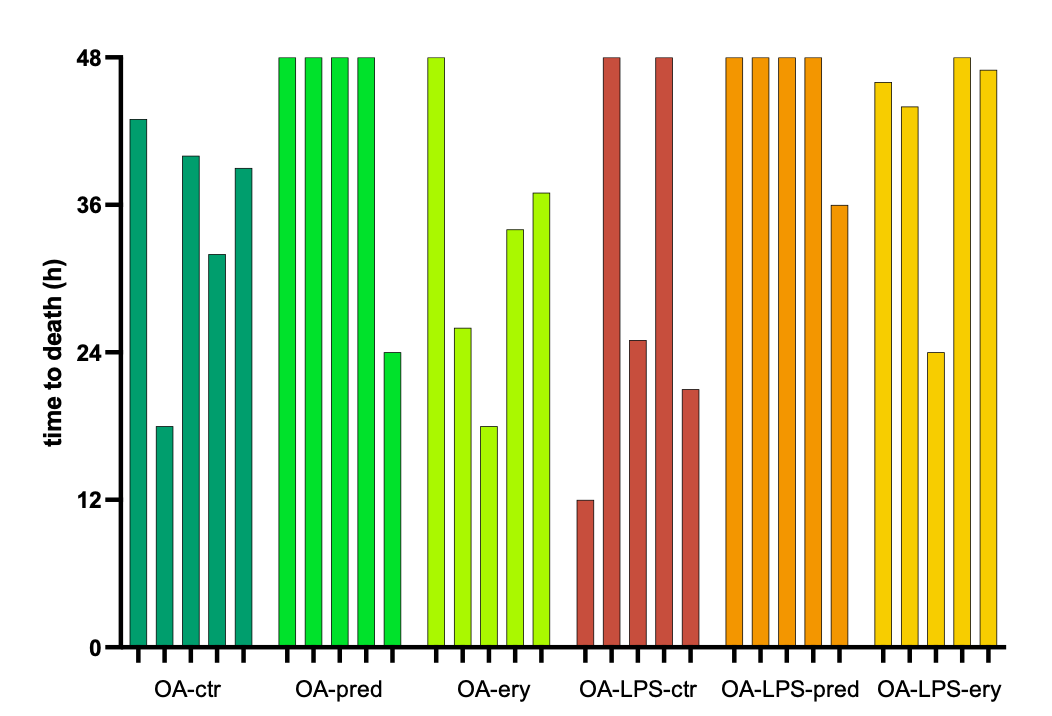
**

Abbreviations: OA = Oleic Acid; OA-LPS = Oleic Acid and lipopolysaccharides; ctr = control; pred = methylprednisolone; ery = erythromycin

**4) Fluid balance and additional hemodynamic parameters: Supplemental Figure S5**

**
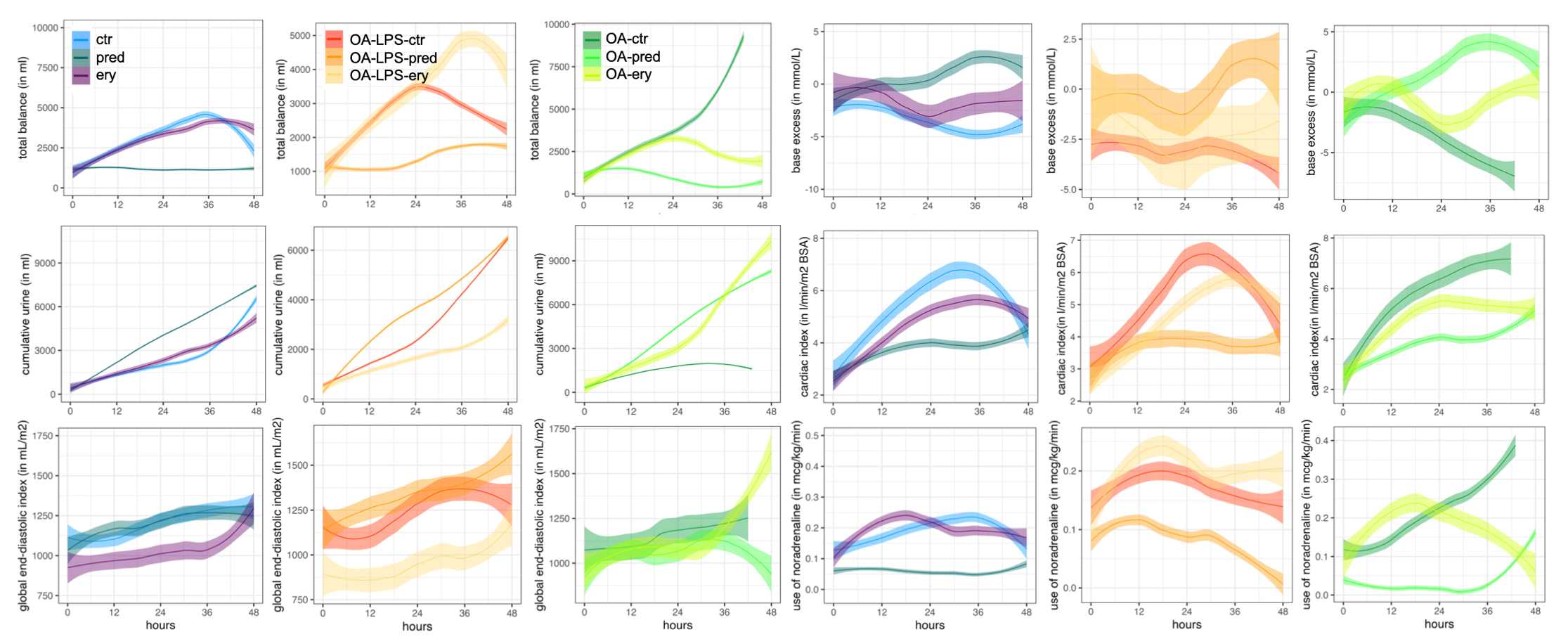
**

Abbreviations: OA = Oleic Acid; OA-LPS = Oleic Acid and lipopolysaccharides; ml = milliliter; mcg = microgram; kg = kilogram; BSA = body surface are

**5) Additional respiratory mechanics parameters: Supplemental Figure S6**

**
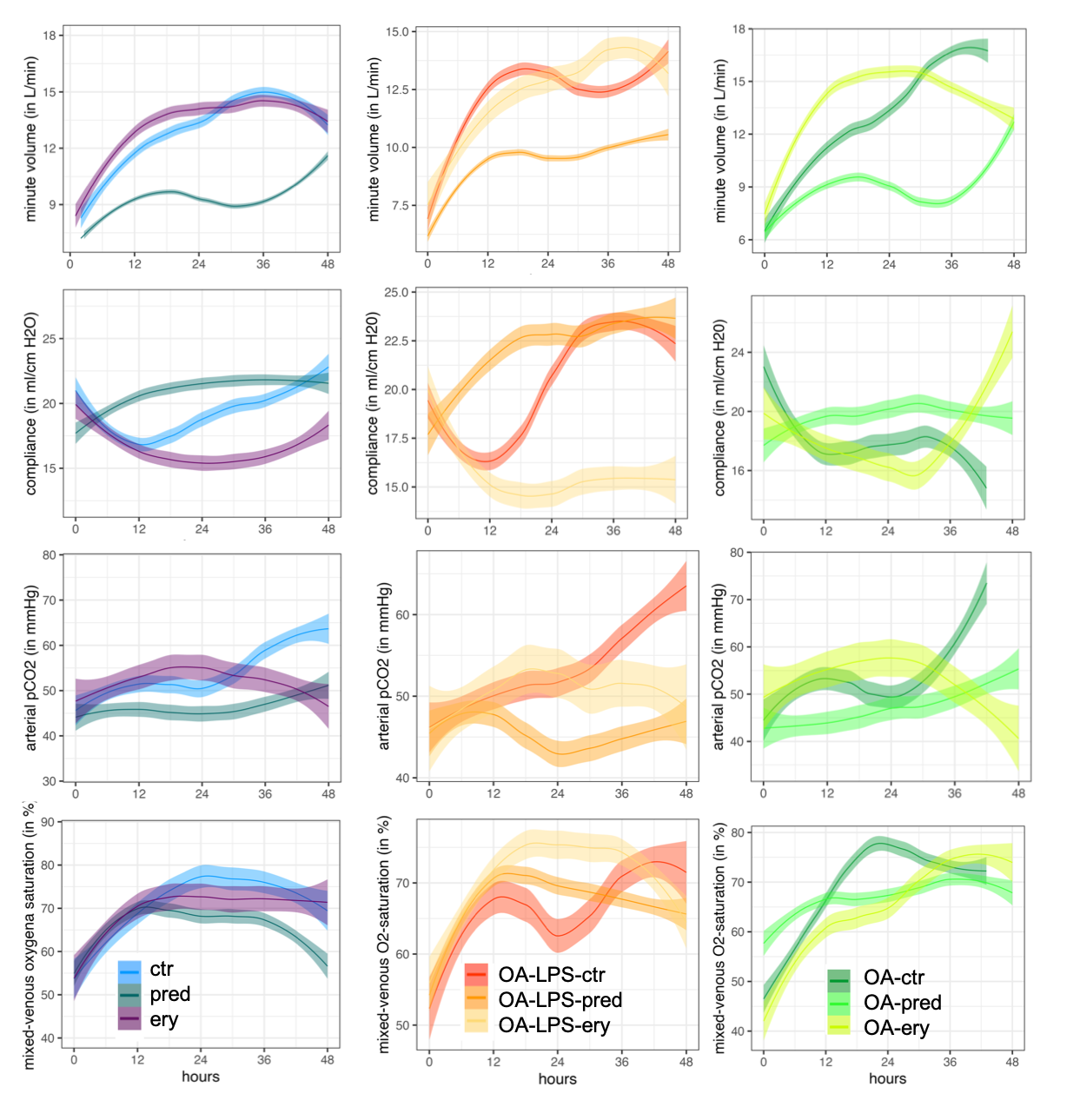
**

Abbreviations: OA = Oleic Acid; OA-LPS = Oleic Acid and lipopolysaccharides; PEEP = Positive end-expiratory pressure; pCO2 = partial pressure of carbon dioxide

**6) Linear mixed-effect model for respiratory mechanics and additional fluid and**

**hemodynamic parameters: Supplemental Table S3**

|  |  | OA and OA-LPS combined | OA-LPS | OA |
| --- | --- | --- | --- | --- |
|  |  | **Estimates (95% CI), p value** | **Estimates (95% CI), p value** | **Estimates (95% CI), p value** |
| Total fluid balance (ml) | | | | |
| Treatment group | Control | Reference | Reference | Reference |
|  | Methylprednisolone | -133 (-1047 to 780), 0.78 | 868 (-2063 to 325), 0.20 | 712 (-592 to 2017), 0.32 |
|  | Erythromycin | -82 (-997 to 832), 0.86 | -557 (-1752 to 634), 0.40 | 574 (-732 to 1880), 0.43 |
| Time, hours |  | 94 (87 to 101), <0.001 | 45 (37 to 54), <0.001 | 145 (136 to 153), <0.001 |
| Interaction between treatment group and time | Control | Reference | Reference | Reference |
|  | Methylprednisolone | -91 (-99 to -82), <0.001 | -24(-34 to -14), <0.001 | -160 (-171 to -149), <0.001 |
|  | Erythromycin | -7 (-17 to 1.7), 0.11 | 51 (40 to 61), <0.001 | -74 (-87 to -61), <0.001 |
| Cumulative urine volume (ml) | | | | |
| Treatment group | Control | Reference | Reference | Reference |
|  | Methylprednisolone | 112 (-845 to 1070), 0.82 | 559 (-450 to 1568), 0.32 | -407 (-1690 to 876), 0.56 |
|  | Erythromycin | 161 (-797 to 1120), 0.75 | 447 (-562 to 1457), 0.42 | -488 (-1773 to 797), 0.49 |
| Time, hours |  | 79 (72 to 85), <0.001 | 109 (103 to 115), <0.001 | 47 (38 to 56), <0.001 |
| Interaction between treatment group and time | Control | Reference | Reference | Reference |
|  | Methylprednisolone | 62 (54 to 70), <0.001 | 8 (0.4 to 15), 0.04 | 119 (108 to 130), <0.001 |
|  | Erythromycin | -5 (-14 to 4), 0.29 | -69 (-77 to -61), <0.001 | 89 (76 to 102), <0.001 |
| Global end-diastolic index (ml/min/m^2^) | | | | |
| Treatment group | Control | Reference | Reference | Reference |
|  | Methylprednisolone | 24 (-131 to 180), 0.77 | 92 (-109 to 293.8), 0.41 | -43(-222 to 136), 0.66 |
|  | Erythromycin | -160 (-316 to -4), 0.06 | -238 (-439 to -37), 0.044 | -93 (-274 to 87), 0.35 |
| Time, hours |  | 3 (1 to 5), 0.007 | 3 (0.4 to 6), 0.031 | 3 (-0.3 to 5.5), 0.082 |
| Interaction between treatment group and time | Control | Reference | Reference | Reference |
|  | Methylprednisolone | 1 (-2 to 4), 0.43 | 3 (-1 to 6), 0.15 | -0.8 (-4.4 to 2.6), 0.64 |
|  | Erythromycin | 1 (-1 to 4), 0.32 | 0.02 (-4 to 4), 0.99 | 4 (-0.5 to 7), 0.08 |
| Base excess (mmol/L) | | | | |
| Treatment group | Control | Reference | Reference | Reference |
|  | Methylprednisolone | 0.5 (-2.0to 2.9), 0.72 | 1.4 (-1.7 to 4.6), 0.41 | -0.6 (-4.3 to 3.1), 0.76 |
|  | Erythromycin | 1.2 (-1.3 to 3.7), 0.35 | 1.3 (-1.9 to 4.4), 0.46 | 1.1 (-2.6 to 4.9), 0.58 |
| Time, hours |  | -0.1 (-0.2 to -0.09), <0.001 | -0.08 (-0.1 to -0.04), <0.001 | -0.2 (-0.2 to -0.1), <0.001 |
| Interaction between treatment group and time | Control | Reference | Reference | Reference |
|  | Methylprednisolone | 0.2 (0.1 to 0.2), <0.001 | 0.1 (0.06 to 0.2), <0.001 | 0.2 (0.2 to 0.3), <0.001 |
|  | Erythromycin | 0.04 (-0.002 to 0.08), 0.064 | 0.006 (-0.05 to 0.06), 0.83 | 0.07 (0.007 to 0.1), 0.03 |
| Cardiac Index (L/min/m^2^ BSA) | | | | |
| Treatment group | Control | Reference | Reference | Reference |
|  | Methylprednisolone | -0.5 (-1.2 to 1.6), 0.15 | -0.4 (-1.6 to 0.8), 0.53 | -0.5 (-1.2 to 0.2), 0.16 |
|  | Erythromycin | -0.6 (-1.3 to 0.05), 0.088 | -1.0 (-2.1 to 0.2), 0.16 | -0.2 (-0.9 to 0.5), 0.60 |
| Time, hours |  | 0.07 (0.06 to 0.08), <0.001 | 0.04 (0.02 to 0.05), <0.001 | 0.1 (0.09 to 0.13), <0.001 |
| Interaction between treatment group and time | Control | Reference | Reference | Reference |
|  | Methylprednisolone | -0.05 (-0.06 to -0.03), <0.001 | -0.02 (-0.04 to -0.003), 0.026 | -0.07 (-0.09 to -0.05), <0.001 |
|  | Erythromycin | -0.001 (-0.01 to 0.02), 0.87 | 0.04 (0.02 to 0.06), <0.001 | -0.03 (-0.06 to -0.01), 0.002 |
| Noradrenaline dosage (mcg/kg/min) | | | | |
| Treatment group | Control | Reference | Reference | Reference |
|  | Methylprednisolone | -0.07 (-0.1 to -0.005), 0.048 | -0.05 (-0.1 to -0.004), 0.31 | -0.08 (-0.2 to -0.004), 0.098 |
|  | Erythromycin | 0.04 (-0.02 to 0.1), 0.21 | -0.009 (-0.08 to 0.09), 0.84 | 0.08 (-0.009 to 0.2), 0.12 |
| Time, hours |  | 0.004 (0.003 to 0.005), <0.001 | 0.002 (0.001 to 0.003), <0.001 | 0.007 (0.006 to 0.008), <0.001 |
| Interaction between treatment group and time | Control | Reference | Reference | Reference |
|  | Methylprednisolone | -0.004 (-0.005 to -0.003), <0.001 | -0.003 (-0.004 to -0.002), <0.001 | -0.005 (-0.006 to -0.004), <0.001 |
|  | Erythromycin | -0.002 (-0.003 to -0.001), <0.001 | -0.0007 (-0.0009 to 0.001), 0.88 | -0.004 (-0.005 to -0.002), <0.001 |
| Minute ventilation (L/min) | | | | |
| Treatment group | Control | Reference | Reference | Reference |
|  | Methylprednisolone | -1.0 (-3.6 to 1.5), 0.44 | -1.9 (-4.8 to 1.0), 0.24 | -0.03 (-4.3 to 4.2), 0.98 |
|  | Erythromycin | 1.1 (-1.5 to 3.6), 0.44 | -1.0 (-3.9 to 1.9), 0.53 | 3.1 (-1.2 to 7.3), 0.19 |
| Time, hours |  | 0.2 (0.17 to 0.2), <0.001 | 0.1 (0.09 to 0.1), <0.001 | 0.3 (0.2 to 0.3), <0.001 |
| Interaction between treatment group and time | Control | Reference | Reference | Reference |
|  | Methylprednisolone | -0.1 (-0.2 to -0.1), <0.001 | -0.06 (-0.09 to -0.03), <0.001 | -0.2 (-0.2 to -0.1), <0.001 |
|  | Erythromycin | -0.01 (-0.03 to 0.01), 0.38 | 0.05 (0.001 to 0.08), 0.005 | -0.06 (-0.1 to -0.02), 0.005 |
| Compliance (ml/cm H_2_0) | | | | |
| Treatment group | Control | Reference | Reference | Reference |
|  | Methylprednisolone | -1.3 (-1.8 to 4.4), 0.41 | 3.1 (-0.7 to 6.9), 0.15 | -0.6 (-5.6 to 4.3), 0.82 |
|  | Erythromycin | -0.6 (-3.7 to 2.6), 0.74 | -0.1 (-3.9 to 3.7), 0.96 | -1.0 (-6.0 to 4.0), 0.72 |
| Time, hours |  | -0.04 (-0.06 to -0.01), 0.002 | 0.07 (0.04 to 0.1), <0.001 | -0.2 (-0.2 to -0.1), <0.001 |
| Interaction between treatment group and time | Control | Reference | Reference | Reference |
|  | Methylprednisolone | 0.08 (0.05 to 0.1), <0.001 | 0.02 (-0.02 to 0.1), 0.41 | 0.1 (0.1 to 0.2), <0.001 |
|  | Erythromycin | -0.02 (-0.06 to 0.01), 0.26 | -0.1 (-0.1 to -0.06), <0.001 | 0.04 (-0.01 to 0.09), 0.11 |
| Arterial CO_2_ partial pressure (mmHg) | | | | |
| Treatment group | Control | Reference | Reference | Reference |
|  | Methylprednisolone | -2.1 (-10.6 to 6.4), 0.64 | 1.2 (-9.4 to 11.8), 0.83 | -5.4 (-19.1 to 8.3), 0.47 |
|  | Erythromycin | 3.1 (-5.4 to 11.4), 0.44 | 1.4 (-9.2 to 12.1), 0.80 | 4.6 (-9.1 to 18.3), 0.54 |
| Time, hours |  | 0.4 (0.3 to 0.5), <0.001 | 0.4 (0.3 to 0.5), <0.001 | 0.5 (0.3 to 0.6), <0.001 |
| Interaction between treatment group and time | Control | Reference | Reference | Reference |
|  | Methylprednisolone | -0.3 (-0.5 to -0.2), <0.001 | -0.5 (-0.6 to -0.3), <0.001 | -0.2 (-0.4 to -0.04), 0.016 |
|  | Erythromycin | -0.2 (-0.3 to -0.03), 0.014 | -0.2 (-0.3 to -0.01), 0.033 | -0.1 (-0.3 to 0.07), 0.24 |
| Mixed-venous oxygen saturation (%) | | | | |
| Treatment group | Control | Reference | Reference | Reference |
|  | Methylprednisolone | 5.8 (-2.9 to 14.4), 0.21 | 4.8 (-7.1 to 16.6), 0.47 | 7.3 (-4.5 to 19.1), 0.27 |
|  | Erythromycin | 0.3 (-8.3 to 9.0), 0.94 | 4.7 (-7.2 to 16.6), 0.48 | -3.8 (-15.7 to 8.0), 0.56 |
| Time, hours |  | 0.3 (0.2 to 0.4), <0.001 | 0.04 (-0.07 to 0.2), 0.51 | 0.5 (0.4 to 0.7), <0.001 |
| Interaction between treatment group and time | Control | Reference | Reference | Reference |
|  | Methylprednisolone | -0.2 (-0.3 to -0.07), 0.001 | 0.06 (-0.08 to 0.2), 0.4 | -0.4 (-0.6 to -0.3), <0.001 |
|  | Erythromycin | 0.07 (-0.04 to 0.2), 0.23 | 0.3 (0.1 to 0.4), <0.001 | -0.1 (-0.3 to 0.07), 0.26 |

**7) Results of full blood count and biochemistry: Supplemental Figure S7**

Overall, there were no major differences in full blood count and biochemistry parameters, except for higher neutrophil count in OA-LPS-pred.

**
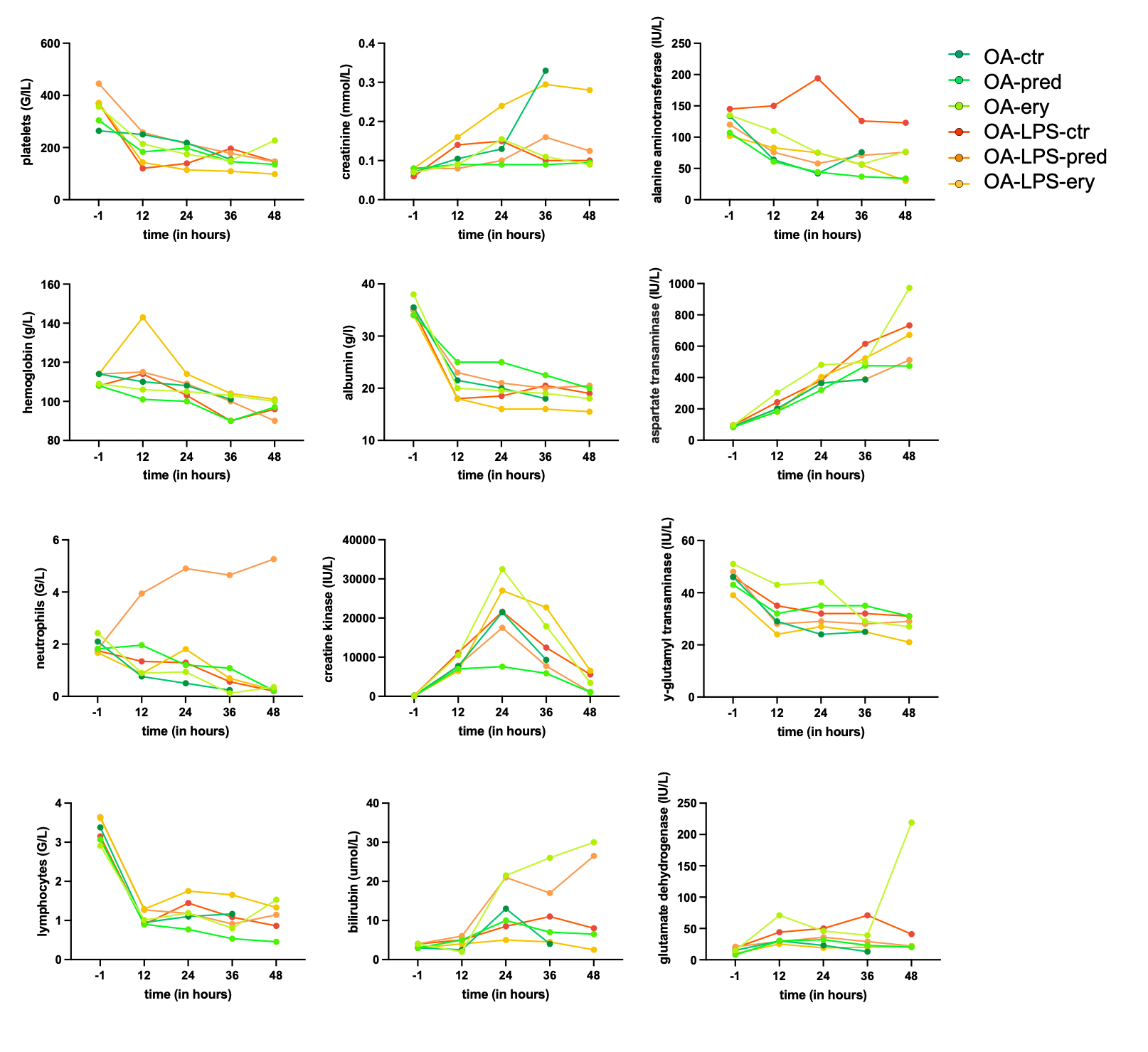
**

Abbreviations: OA = Oleic Acid; OA-LPS = Oleic Acid and LPS; ctr = control; methyl = methylprednisolone; ery = erythromycin

**8) Cumulative survival among A) treatment groups (injury types combined) B) treatment among OA-LPS and C) treatment among OA: Supplemental Figure S8**

**
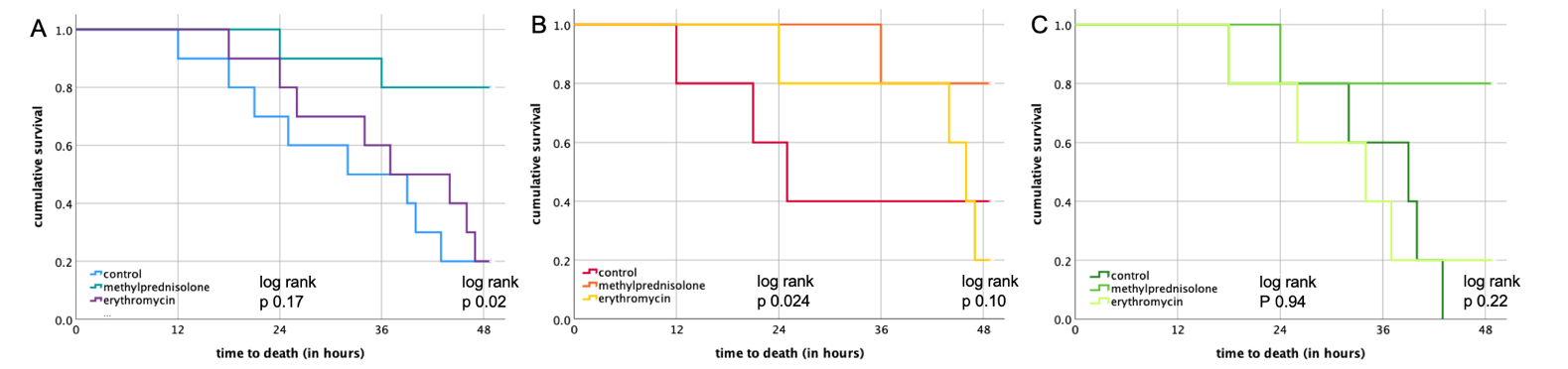
**Abbreviations: OA = Oleic Acid; OA-LPS = Oleic Acid and lipopolysaccharides

**9) Lung Injury Score among deceased and surviving animals: Supplemental Figure S8**

**
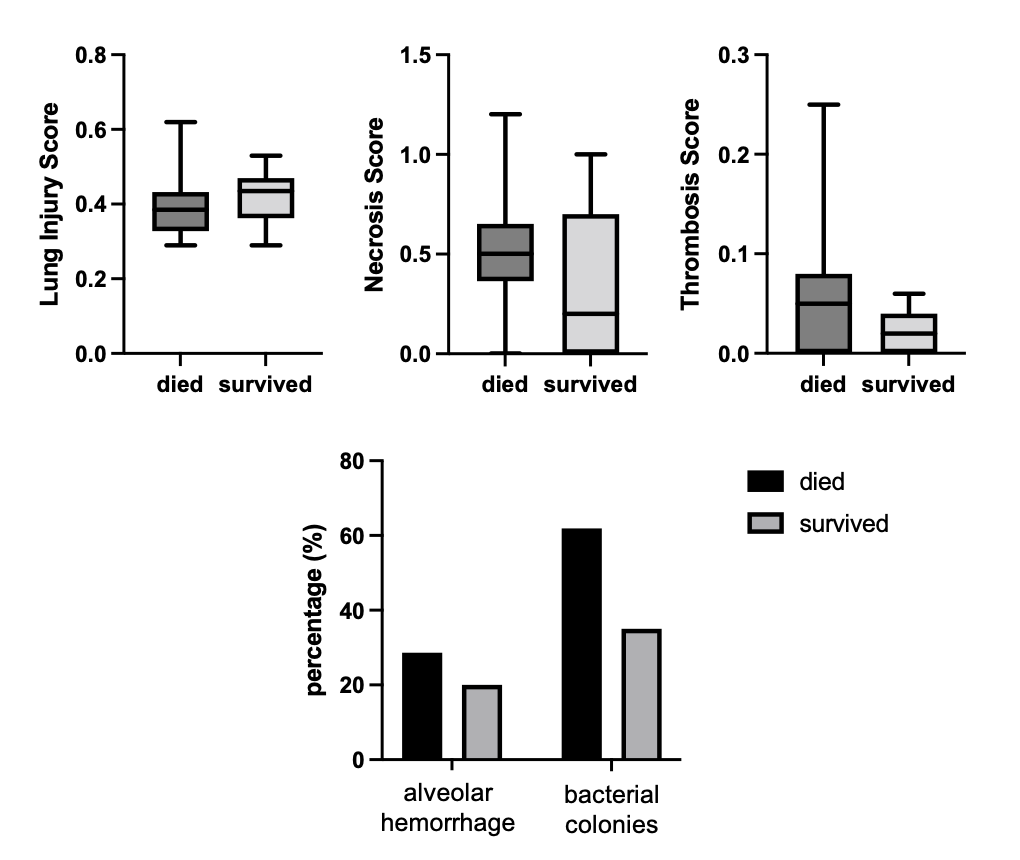
**

**10) Bayesian model for predictors of survival and treatment effect: Supplemental Figure S10**

The Bayesian joint model for parameter estimates and treatment effect displays A) hazard ratio for the assessed parameter and B) hazard ratio for the treatment effect among OA and OA-LPS


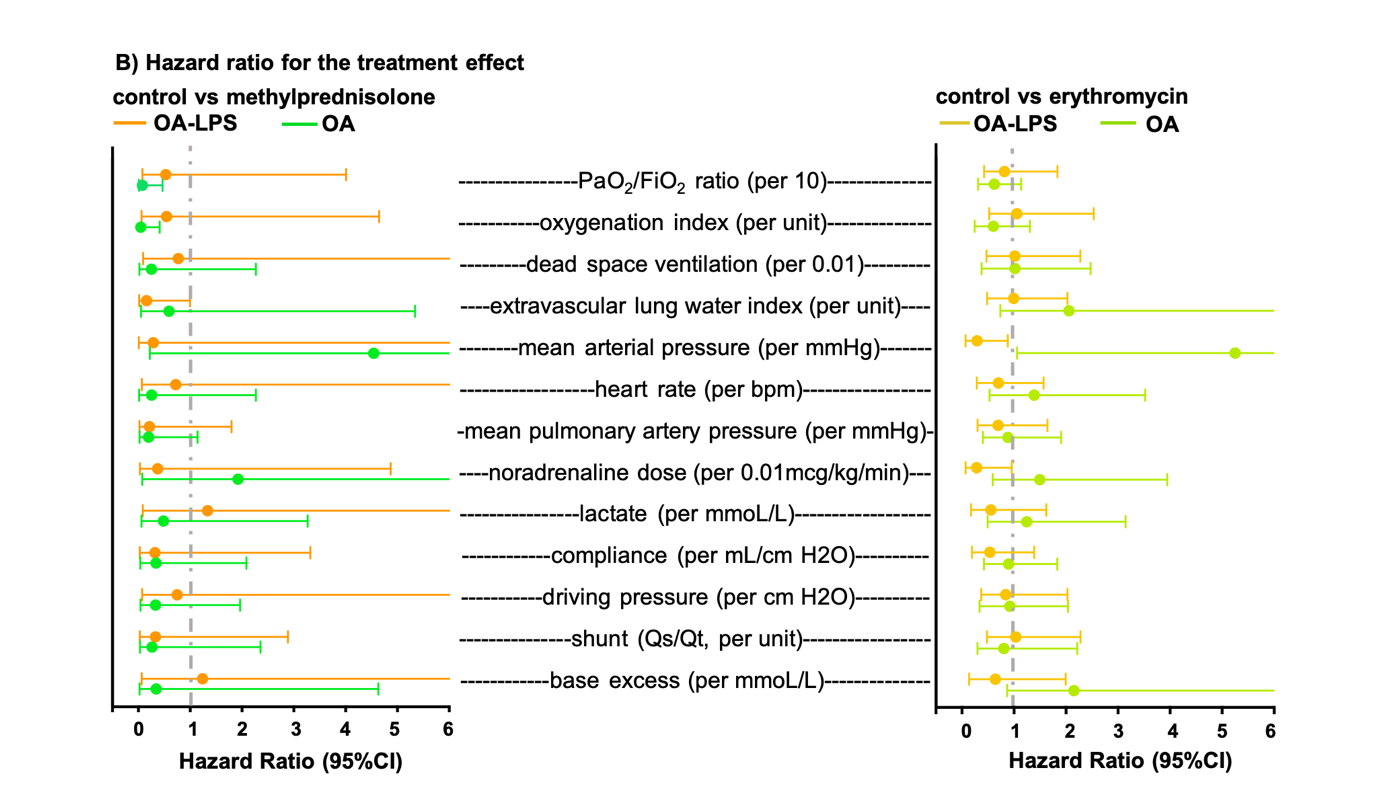

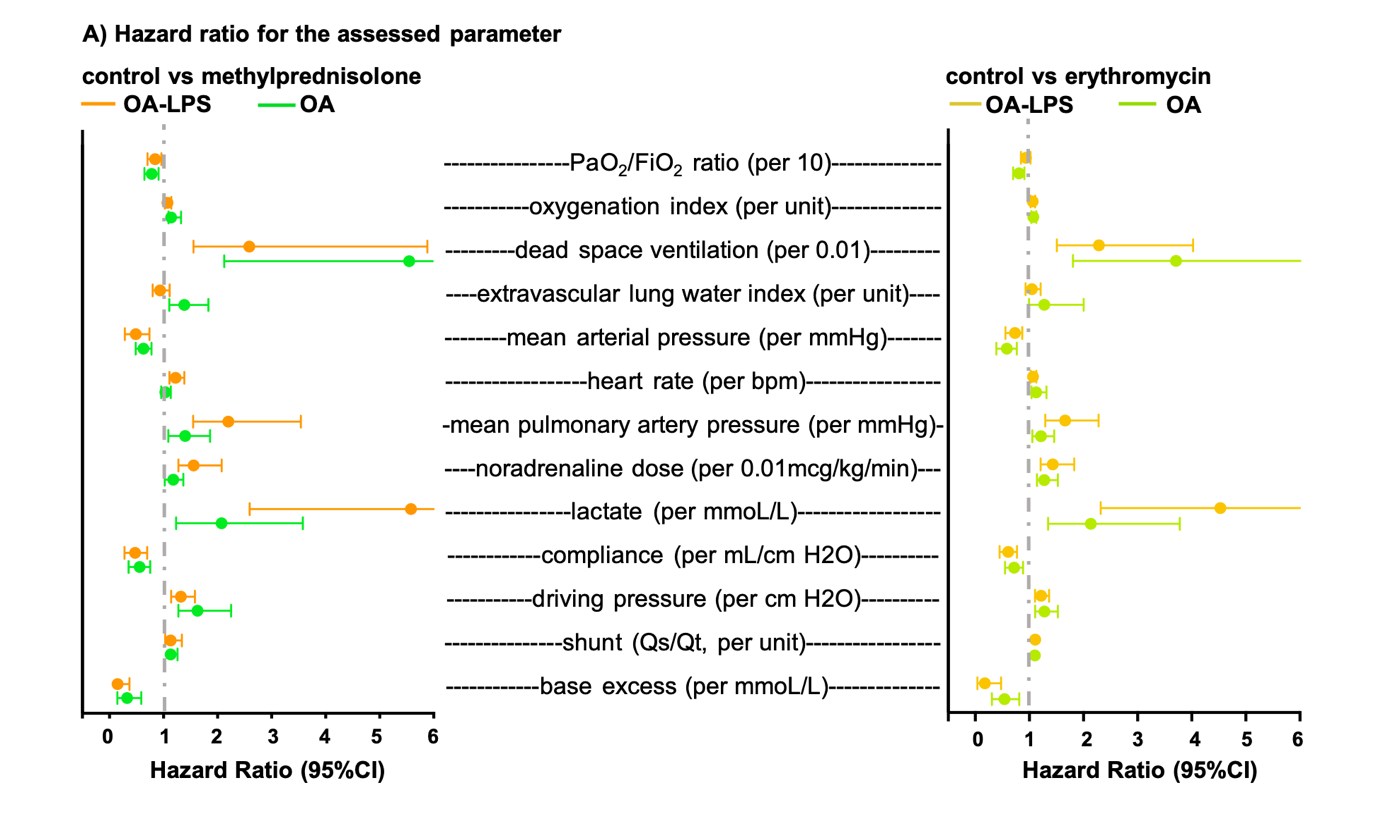


Abbreviations: bpm = beat per minute; CI = confidence interval; OA = Oleic Acid; OA-LPS = Oleic Acid and LPS; Qs/Qt = shunt

**11) Model specification and detailed results of Bayesian joint model for survival and treatment effect: Supplemental Table S4A-D**

A) OA-LPS: controls vs treatment with methylprednisolone

|  | **Longitudinal measurement** | | | **Treatment effect** | | |
| --- | --- | --- | --- | --- | --- | --- |
|  | HR (95%CI) | rhat | mcse | HR (95%CI) | rhat | mcse |
| PaO_2_/FiO_2_ ratio (per 10) | 0.843 (0.703-0.960) | 1.001 | 0.004 | 0.529 (0.080-4.01) | 0.999 | 0.038 |
| Oxygenation index (per unit) | 1.073 (1.026-1.143) | 1 | 0.001 | 0.544 (0.06-4.647) | 0.999 | 0.047 |
| Dead space ventilation (per 0.01) | 2.588 (1.553-5.883) | 0.999 | 0.015 | 0.771 (0.091-6.209) | 0.999 | 0.036 |
| Ventilatory Ratio (per 1) | 1.857 (0.889-4.579) | 1.004 | 0.015 | 0.620 (0.066-5.855) | 1 | 0.043 |
| Extravascular lung water index (per unit) | 0.937 (0.799-1.112) | 0.999 | 0.003 | 0.159 (0.016-0.997) | 1 | 0.047 |
| Mean arterial pressure (per mmHg) | 0.487 (0.286-0.739) | 0.999 | 0.009 | 0.289 (0.008-7.933) | 1 | 0.059 |
| Heart rate (per bpm) | 1.225 (1.109-1.386) | 0.999 | 0.002 | 0.721 (0.066-6.919) | 1 | 0.039 |
| Mean pulmonary artery pressure (per mmHg) | 2.201 (1.547-3.542) | 0.999 | 0.009 | 0.214 (0.023-1.799) | 0.999 | 0.041 |
| Noradrenaline dose (per 0.01ug/kg/min) | 1.558 (1.275-2.076) | 1.008 | 0.006 | 0.373 (0.030-4.870) | 0.999 | 0.047 |
| Lactate (per mmoL/L) | 5.578 (2.593-16.653) | 1 | 0.022 | 1.338 (0.085-21.64) | 0.999 | 0.053 |
| Compliance (per mL/cm H_2_0) | 0.473 (0.28-0.698) | 1 | 0.011 | 0.321 (0.029-3.318) | 0.999 | 0.044 |
| Driving pressure (per cm H_2_0) | 1.323 (1.141-1.582) | 0.999 | 0.003 | 0.750 (0.074-8.137) | 0.999 | 0.043 |
| Shunt (Qs/Qt; per unit) | 1.131 (1.031-1.340) | 1.014 | 0.005 | 0.330 (0.027-2.889) | 1 | 0.044 |
| Base excess (per mmoL/L) | 0.151 (0.052-0.371) | 1 | 0.025 | 1.240 (0.065-16.495) | 0.999 | 0.051 |

B) OA-LPS: controls vs treatment with erythromycin

|  | **Longitudinal measurement** | | | **Treatment effect** | | |
| --- | --- | --- | --- | --- | --- | --- |
|  | HR (95%CI) | rhat | mcse | HR (95%CI) | rhat | mcse |
| PaO_2_/FiO_2_ ratio (per 10) | 0.935 (0.844-1.023) | 1 | 0.002 | 0.816 (0.425-1.834) | 1 | 0.014 |
| Oxygenation index (per unit) | 1.062 (1.022-1.107) | 1 | 0.001 | 1.055 (0.523-2.533) | 1 | 0.015 |
| Dead space ventilation (per 0.01) | 2.285 (1.508-4.027) | 0.999 | 0.01 | 1.018 (0.472-2.277) | 0.999 | 0.003 |
| Ventilatory Ratio (per 1) | 1.511 (1.078-2.167) | 1.003 | 0.007 | 1.153 (0.573-2.356) | 1.001 | 0.012 |
| Extravascular lung water index (per unit) | 1.045 (0.931-1.209) | 0.999 | 0.002 | 0.993 (0.482-2.029) | 0.999 | 0.012 |
| Mean arterial pressure (per mmHg) | 0.734 (0.556-0.87) | 0.999 | 0.007 | 0.290 (0.069-0.880) | 0.999 | 0.036 |
| Heart rate (per bpm) | 1.069 (1.029-1.128) | 0.999 | 0.001 | 0.702 (0.284-1.570) | 1 | 0.016 |
| Mean pulmonary artery pressure (per mmHg) | 1.662 (1.295-2.278) | 1.001 | 0.006 | 0.693 (0.299-1.643) | 0.999 | 0.014 |
| Noradrenaline dose (per 0.01ug/kg/min) | 1.432 (1.207-1.829) | 0.999 | 0.005 | 0.282 (0.069-0.959) | 0.999 | 0.033 |
| Lactate (per mmoL/L) | 4.529 (2.319-12.566) | 0.999 | 0.026 | 0.557 (0.173-1.621) | 0.999 | 0.025 |
| Compliance (per mL/cm H_2_0) | 0.608 (0.45-0.77) | 1.001 | 0.006 | 0.534 (0.193-1.390) | 1 | 0.019 |
| Driving pressure (per cm H_2_0) | 1.219 (1.103-1.363) | 0.999 | 0.002 | 0.842 (0.37-2.027) | 0.999 | 0.015 |
| Shunt (Qs/Qt; per unit) | 1.106 (1.041-1.187) | 1.002 | 0.001 | 1.035 (0.481-2.278) | 0.999 | 0.013 |
| Base excess (per mmoL/L) | 0.179 (0.039-0.477) | 1 | 0.046 | 0.642 (0.137-1.994) | 1 | 0.036 |

C) OA: controls vs treatment with methylprednisolone

|  | **Longitudinal measurement** | | | **Treatment effect** | | |
| --- | --- | --- | --- | --- | --- | --- |
|  | HR (95%CI) | rhat | mcse | HR (95%CI) | rhat | mcse |
| PaO_2_/FiO_2_ ratio (per 10) | 0.786 (0.651-0.914) | 0.999 | 0.004 | 0.081 (0.011-0.468) | 1 | 0.039 |
| Oxygenation index (per unit) | 1.148 (1.098-1.33) | 1.005 | 0.004 | 0.049 (0.011-0.414) | 1.003 | 0.056 |
| Dead space ventilation (per 0.01) | 5.564 (2.132-21.801) | 1.002 | 0.027 | 0.254 (0.0217-2.274) | 1.003 | 0.05 |
| Ventilatory Ratio (per 1) | 1.844 (1.181-3.014) | 1 | 0.012 | 0.539 (0.052-3.756) | 0.999 | 0.05 |
| Extravascular lung water index (per unit) | 1.392 (1.112-1.838) | 0.999 | 0.006 | 0.593 (0.053-5.347) | 0.999 | 0.044 |
| Mean arterial pressure (per mmHg) | 0.634 (0.491-0.784) | 1.002 | 0.007 | 4.55 (0.224-73.643) | 0.999 | 0.059 |
| Heart rate (per bpm) | 1.041 (0.965-1.14) | 0.999 | 0.001 | 0.258 (0.016-2.273) | 0.999 | 0.043 |
| Mean pulmonary artery pressure (per mmHg) | 1.408 (1.096-1.869) | 0.999 | 0.005 | 0.199 (0.024-1.143) | 0.999 | 0.033 |
| Noradrenaline dose (per 0.01ug/kg/min) | 1.186 (1.027-1.373) | 0.999 | 0.003 | 1.927 (0.076-51.507) | 0.999 | 0.058 |
| Lactate (per mmoL/L) | 2.083 (1.239-3.592) | 0.999 | 0.013 | 0.485 (0.062-3.273) | 0.999 | 0.045 |
| Compliance (per mL/cm H_2_0) | 0.563 (0.359-0.76) | 0.999 | 0.008 | 0.342 (0.04-2.089) | 1.005 | 0.034 |
| Driving pressure (per cm H_2_0) | 1.637 (1.282-2.261) | 1.001 | 0.006 | 0.334 (0.043-1.966) | 0.999 | 0.045 |
| Shunt (Qs/Qt; per unit) | 1.139 (1.071-1.265) | 1 | 0.002 | 0.267 (0.035-2.362) | 1 | 0.06 |
| Base excess (per mmoL/L) | 0.331 (0.15-0.595) | 1.001 | 0.02 | 0.345 (0.024-4.637) | 0.999 | 0.05 |

D) OA: controls vs treatment with erythromycin

|  | **Longitudinal measurement** | | | **Treatment effect** | | |
| --- | --- | --- | --- | --- | --- | --- |
|  | HR (95%CI) | rhat | mcse | HR (95%CI) | rhat | mcse |
| PaO_2_/FiO_2_ ratio (per 10) | 0.809 (0.698-0.907) | 1.001 | 0.003 | 0.623 (0.313-1.142) | 0.999 | 0.013 |
| Oxygenation index (per unit) | 1.072 (1.035-1.125) | 0.999 | 0.001 | 0.606 (0.245-1.309) | 1 | 0.015 |
| Dead space ventilation (per 0.01) | 3.703 (1.802-10.330) | 1.002 | 0.019 | 1.021 (0.378-2.478) | 0.999 | 0.015 |
| Ventilatory Ratio (per 1) | 2.165 (1.444-3.786) | 1 | 0.009 | 1.704 (0.650-5.267) | 0.999 | 0.016 |
| Extravascular lung water index (per unit) | 1.274 (0.991-2.001) | 0.999 | 0.008 | 2.063 (0.740-11.397) | 0.999 | 0.027 |
| Mean arterial pressure (per mmHg) | 0.58 (0.390-0.766) | 0.999 | 0.01 | 5.257 (1.062-38.543) | 1.002 | 0.035 |
| Heart rate (per bpm) | 1.123 (1.037-1.311) | 1 | 0.002 | 1.393 (0.533-3.527) | 0.999 | 0.014 |
| Mean pulmonary artery pressure (per mmHg) | 1.221 (1.051-1.455) | 0.999 | 0.002 | 0.887 (0.404-1.908) | 0.999 | 0.011 |
| Noradrenaline dose (per 0.01ug/kg/min) | 1.274 (1.138-1.524) | 1.003 | 0.004 | 1.50 (0.594-3.953) | 1.008 | 0.019 |
| Lactate (per mmoL/L) | 2.132 (1.344-3.776) | 1 | 0.009 | 1.248 (0.497-3.151) | 1 | 0.015 |
| Compliance (per mL/cm H_2_0) | 0.717 (0.548-0.881) | 0.999 | 0.003 | 0.899 (0.425-1.834) | 0.999 | 0.012 |
| Driving pressure (per cm H_2_0) | 1.276 (1.105-1.524) | 1 | 0.003 | 0.924 (0.339-2.044) | 1 | 0.015 |
| Shunt (Qs/Qt; per unit) | 1.097 (1.042-1.192) | 0.999 | 0.001 | 0.808 (0.301-2.220) | 1.001 | 0.018 |
| Base excess (per mmoL/L) | 0.539 (0.305-0.811) | 0.999 | 0.009 | 2.157 (0.873-6.746) | 0.999 | 0.017 |

Abbreviations: OA = Oleic Acid; OA-LPS = Oleic Acid and lipopolysaccharides; 95%CI = 95% credible intervals; HR = hazard ratio; mcse = monte carlo standard error; rhat = Potential Scale Reduction Factor by Gelman and Rubin

The shared parameter joint model incorporates two linked submodels: the longitudinal submodel (patterns of change in a variable measured repeatedly over time) and the time-to-event submodel. The additional tying-in of the association between these 2 submodels results in a reduction in estimation bias, an increased efficiency and offers the option to predict outcomes based on the parameter distribution.

The Bayesian approach estimates the joint posterior distribution of the model that consists of the joint likelihood of the longitudinal and time-to-event outcome and the joint prior distribution. Prior information from f.e. previous studies result in a so called “informative distribution”, generally allowing a dynamic prediction by incorporating new longitudinal information in the model. If there is no prior information or there is no need to adjust the model fit with any prior information, the prior distribution is called non-informative.

The method used for drawing samples from the posterior distribution for the estimation of the parameters is the Markov chain Monte Carlo (MCMC) sampling algorithm, the most commonly used. The algorithm constructs a Markov chain in numerous steps (iterations) that approaches the desired distribution (its stationary) as the number of iterations increases. The sample is drawn from the state of the chain after a defined number of steps. The Monte Carlo Standard Error (MSCE) is displayed as the parameter of accuracy of the MCMC chains, it is calculated as the standard deviation of the chains divided by their effective sample size. Lower MCSE indicate more accurate estimations of the true means. An indicator of the assessment of convergence of the MCMC is given by the Rhat (potential scale reduction factor on split chains): a chain has converged when stationary is reached (all samples display the same distribution). The Rhat displays the comparison of the between- and within-chain estimates for model parameters, therefore a Rhat close to 1 indicates a well converged chain.

**Supplemental references**

1. Percie du Sert N, Ahluwalia A, Alam S, Avey MT, Baker M, Browne WJ, et al. Reporting animal research: Explanation and elaboration for the ARRIVE guidelines 2.0. PLOS Biol. 2020 Jul 14;18(7):e3000411.

2. Mercat A, Richard J-CM, Vielle B, Jaber S, Osman D, Diehl J-L, et al. Positive end-expiratory pressure setting in adults with acute lung injury and acute respiratory distress syndrome: a randomized controlled trial. J Am Med Assoc. 2008 Feb;299(6):646–55.

3. Bennett JW. Regional body surface area of sheep. J Agric Sci. 1973 Dec 27;81(3):429–32.

4. Bouquet M, Passmore MR, See Hoe LE, Tung J-P, Simonova G, Boon A-C, et al. Development and validation of ELISAs for the quantitation of interleukin (IL)-1β, IL-6, IL-8 and IL-10 in ovine plasma. J Immunol Methods. 2020 Nov;486:112835.

5. Matute-Bello G, Downey G, Moore BB, Groshong SD, Matthay MA, Slutsky AS, et al. An Official American Thoracic Society Workshop Report: Features and Measurements of Experimental Acute Lung Injury in Animals. Am J Resp Cell Mol Biol. 2011;44(5):725–38.

6. Trachsel D, McCrindle BW, Nakagawa S, Bohn D. Oxygenation Index Predicts Outcome in Children with Acute Hypoxemic Respiratory Failure. Am J Respir Crit Care Med. 2005 Jul 15;172(2):206–11.

7. Ferluga M, Lucangelo U, Blanch L. Dead space in acute respiratory distress syndrome. Ann Transl Med. 2018 Oct;6(19):388–93. A

8. Leigh L, Tyrell M, Strickland D. Simplified versions of the shunt and oxygen consumption equation. Anesthesiology. 1969;30(4):468–70.

9. Cruz DN, Antonelli M, Fumagalli R, Foltran F, Brienza N, Donati A, et al. Early Use of Polymyxin B Hemoperfusion in Abdominal Septic Shock. J Am Med Assoc. 2009 Jun 17;301(23):2445.
